# Phosphorylation of Ephexin4 at Ser-41 contributes to chromosome alignment *via* RhoG activation in cell division

**DOI:** 10.1016/j.jbc.2024.108084

**Published:** 2024-12-13

**Authors:** Ryuji Yasutake, Hiroki Kuwajima, Ryuzaburo Yuki, Junna Tanaka, Youhei Saito, Yuji Nakayama

**Affiliations:** Laboratory of Biochemistry and Molecular Biology, Kyoto Pharmaceutical University, Kyoto, Japan

**Keywords:** Ephexin4, GEF, RhoG, EphA2, phosphorylation, chromosome misalignment

## Abstract

Ephexin proteins are guanine nucleotide exchange factors for the Rho GTPases. We reported that Ephexin4 regulates M-phase progression downstream of phosphorylated EphA2, a receptor-type tyrosine kinase, through RhoG activation; however, the regulation of Ephexin4 during M phase remains unknown. In this study, a novel Ephexin4 phosphorylation site was identified at Ser41, exclusively in M phase. Ephexin4 knockdown prolonged the duration of M phase by activating the spindle assembly checkpoint, at which BubR1 was localized at the kinetochores of the misaligned chromosomes. This delay was alleviated by re-expression of wild-type, but not S41A Ephexin4. The Ephexin4 knockdown caused chromosome misalignment and reduced the RhoG localization to the plasma membrane. These phenotypes were rescued by re-expression of wild type and phospho-mimic S41E mutant, but not the S41A mutant. Consistently, S41E mutant enhanced active RhoG levels, even in the interphase. Regardless of the Ephexin4 knockdown, active RhoG-G12V was localized at the plasma membrane. Furthermore, Ephexin4 knockdown exacerbated vincristine-induced chromosome misalignment, which was prevented by re-expressing the wild-type but not S41A Ephexin4. Overexpression of wild type and S41E mutant, but not S41A mutant, resulted in an increased number of Madin–Darby canine kidney cysts with cells inside the lumen, indicating disruption of epithelial morphogenesis by deregulating Ephexin4/RhoG signaling in cell division. Our results suggest that Ephexin4 undergoes phosphorylation at Ser41 in cell division, and the phosphorylation is required for chromosome alignment through RhoG activation. Combined with mitosis-targeting agents, inhibition of Ephexin4 phosphorylation may represent a novel strategy for cancer chemotherapy.

Eph-interacting exchange protein (Ephexin) family proteins are guanine nucleotide exchange factors (GEFs) of Rho GTPases, in which they catalyze the exchange of bound GDP for GTP. Ephexins are involved in a variety of cellular processes, including cytoskeleton regulation, motility, cell proliferation, and tumorigenesis, by enabling Rho GTPases to interact with their downstream proteins. Ephexins contain a Dbl homology (DH) domain and an adjacent pleckstrin homology (PH) domain, both of which are characteristic of the Dbl family proteins and responsible for the catalytic activity of GEFs. Ephexins act as downstream of Eph receptors, which are the largest subfamily of tyrosine kinase receptors (1). Ephexin4, a member of Ephexin family and also known as ARHGEF16, functions as a RhoG-GEF downstream of the EphA2 signal (2).

We previously reported that Ephexin4 regulates M-phase progression downstream of EphA2 (3, 4). Ephexin4 is recruited to phosphorylated EphA2 at Ser897 in M phase as well as interphase; however, EphA2 phosphorylation is catalyzed by the CDK1–MEK–ERK–RSK pathway in M phase in a different manner compared with interphase. Upon recruitment to phosphorylated EphA2, Ephexin4 promotes the plasma membrane localization of RhoG. EphA2 knockdown delays M-phase progression, which is rescued by the expression of the active RhoG-G12V mutant. Therefore, the EphA2–Ephexin4–RhoG axis plays a role in M-phase progression by regulating proper cortical actin formation. Cortical actin is required for spindle formation and its positioning (5) and for mitotic cell rounding, which contributes to chromosome alignment at the spindle equator (6). Therefore, Ephexin4 participates in the regulation of cell division through cortex regulation; however, the manner in which Ephexin4 activity is regulated in M phase is unknown.

The activities of the Ephexins are inhibited by intra- and inter-molecular interactions that involve the N-terminal region (7, 8, 9, 10, 11). Tyrosine phosphorylation of the inhibitory helix in the N-terminal half by Src tyrosine kinase prevents these interactions and results in GEF activation of Ephexin1–Ephexin3 (7, 8, 12). However, Ephexin4 is not phosphorylated by Src (13); the signal to activate Ephexin4 is unclear. Using the PhosphoSitePlus (https://www.phosphosite.org/) database, we discovered that several phosphorylation sites exist in the N-terminal half of Ephexin4. In this study, to explore its regulatory mechanism in M phase, we focused on phosphorylation in the N-terminal half of Ephexin4. We identified Ser41 phosphorylation in M phase, and the phosphorylation is necessary for chromosome alignment at the mitotic spindle equator through RhoG activation. Ephexin4 is significantly increased in various cancer cells, based on TCGA dataset analysis, and promotes cell migration, invasion, and colony formation (2, 14). Notably, cyst formation assay showed that Ephexin4 overexpression resulted in the disruption of epithelial morphogenesis in a manner dependent on Ser41 phosphorylation. We further demonstrated that inhibition of Ser41 phosphorylation potentiates sensitivity to the anticancer agent vincristine. Our findings provide the possibility of involvement of Ser41 phosphorylation in the oncogenesis of overexpressed Ephexin4 and a preclinical theory for the application of kinase inhibition of Ephexin4 phosphorylation to induce a severe defect in M-phase, thus causing cell death.

## Results

### Ephexin4 is involved in the regulation of M-phase progression

**Figure 1:**
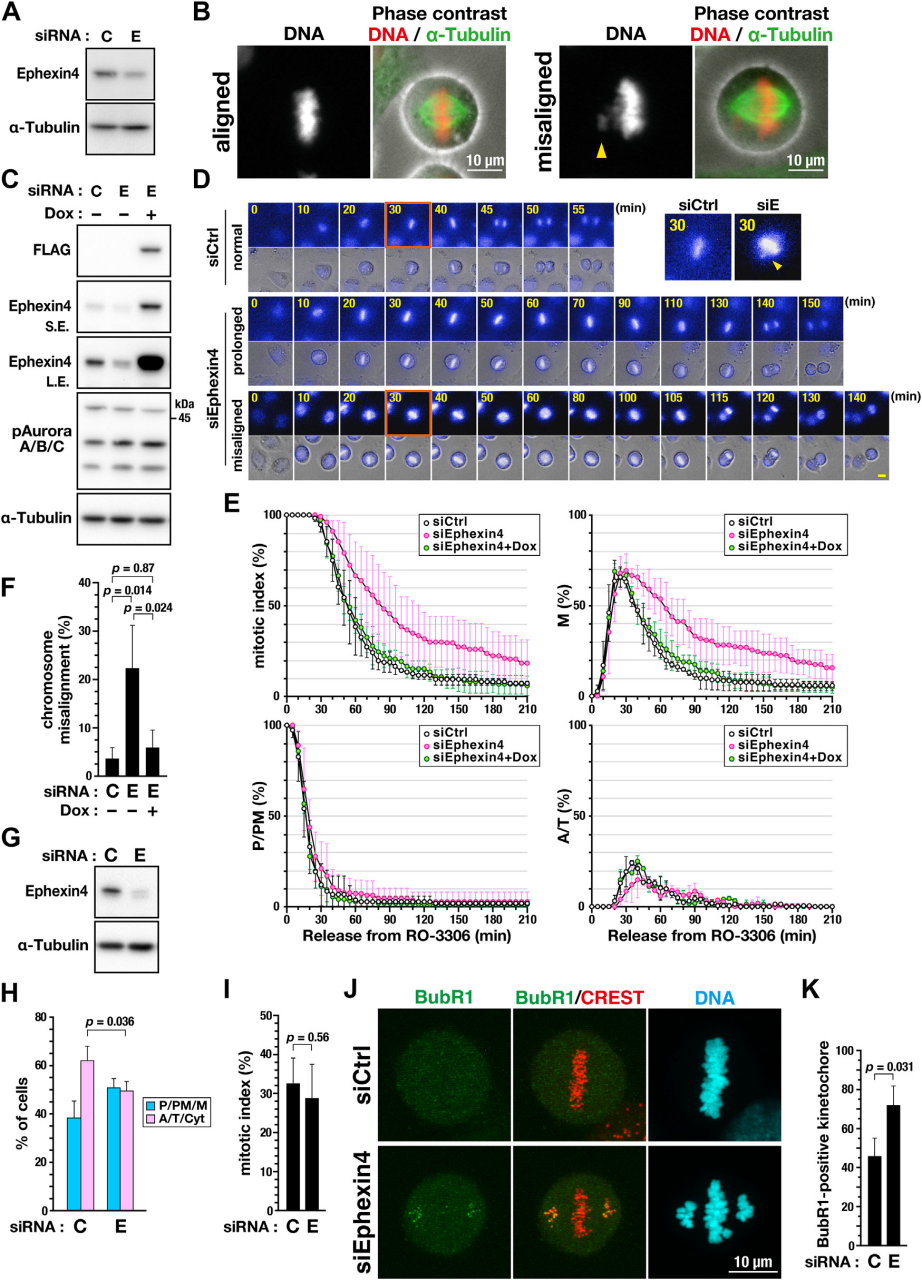
**Ephexin4 knockdown delays M-phase progression.***A* and *B*, HeLa S3 cells were transfected with nontargeting control (C, 100 nM) or Ephexin4-targeting (E, #3, 100 nM) siRNA and cultured for 48 h. Whole-cell lysates were obtained and subjected to Western blot (WB) analysis (*A*). The cells were fixed and stained for DNA and α-tubulin. Representative images of a cell at normal metaphase and with misaligned chromosomes are shown (*B*). *C*, HeLa S3/WT cells were transfected with Ephexin4-targeting (E, #3, 250 nM) or nontargeting (C, 250 nM) siRNAs. After 7.5 h of transfection, the cells were treated with 4 mM thymidine for 24 h. After a 6-h release from thymidine, the cells were treated with 6 μM RO-3306 for 10.5 h, followed by incubation in RO-3306–free medium for 30 min. The cells were then cultured in the presence of 40 μM MG132 for 90 min, and whole-cell lysates of M-phase cells were subjected to WB analysis. For Dox-inducible expression, cells were continuously treated with 90 ng/ml Dox from the siRNA transfection to RO-3306 treatment. *D*–*F*, HeLa S3/WT cells were transfected with Ephexin4-targeting (siEphexin4, 250 nM) or nontargeting (siCtrl, 250 nM) siRNA, and after a 28-h incubation, the cells were treated with 6 μM RO-3306 for 20 h. For WT Ephexin4 re-expression, the cells were cultured with medium containing 90 ng/ml Dox during siRNA treatment until the end of RO-3306 treatment. Immediately after release from RO-3306, the cells were monitored every 5 min in the presence of 0.1 μM Hoechst 33342 for 4 h by time-lapse imaging. Representative images of cells showing normal and prolonged M-phase progression and chromosome misalignment are shown. Cells indicated by *orange squares* are magnified and shown on the *right*. Arrowheads designate misaligned chromosomes. Scale bar represents 10 μm (*D*). The duration of each mitotic subphase of each cell is shown in Fig. S1. The percentages of M-phase (mitotic index), prophase/prometaphase (P/PM), metaphase (M), and anaphase/telophase (A/T) cells are plotted at each time point as the mean ± SD calculated from three independent experiments (*E*). The percentage of cells with chromosome misalignment is shown as the mean ± SD calculated from three independent experiments. (n ≥ 32 cells per condition). Tukey’s test was used to determine *p*-values (*F*). *G*, A549 cells were transfected with Ephexin4-targeting (E, #2, 50 nM) or nontargeting (C, 50 nM) siRNA and cultured for 48 h. Whole-cell lysates were prepared and subjected to WB analysis. *H* and *I*, A549 cells were treated with Ephexin4-targeting (E, #2, 50 nM) or nontargeting (C, 50 nM) siRNA for 48 h. During the last 20 h, the cells were treated with 5 μM RO-3306. After a 50-min release from RO-3306, the cells were fixed and stained for DNA and α-tubulin. The cells were classified into two groups before (P/PM/M) and after (A/T/C) the onset of anaphase based on DNA and microtubule morphologies. The percentage of the two groups (*H*; n ≥ 256 cells per condition) and mitotic index (*I*; n ≥ 1011 cells per condition) are shown as the mean ± SD calculated from three independent experiments. A Student’s *t* test was used to determine *p*-values. *J* and *K*, HeLa S3 cells were transfected with Ephexin4-targeting (siEphexin4, 250 nM) or nontargeting (siCtrl, 250 nM) siRNA, and after a 28-h incubation, the cells were treated with 6 μM RO-3306 for 20 h. Then, the cells were cultured for 30 min without RO-3306, followed by a 60-min incubation with 40 μM MG132. Then, the cells were fixed and stained for BubR1, CREST, and DNA. Representative images are shown in *J*. Scale bar represents 10 μm. In *K*, the percentage of metaphase cells with BubR1-positive kinetochore is plotted as the mean ± SD calculated from three independent experiments (n ≥ 50 per condition). A Student’s *t* test was used to determine *p*-values.

To examine the role of Ephexin4 in cell division, Ephexin4 was knocked down by Ephexin4-targeting (E, #3) siRNA (Fig. 1*A*). Despite the typical alignment of most chromosomes on the metaphase plate, a few chromosomes were frequently misaligned (Fig. 1*B*, misaligned, yellow arrowhead), confirming a role of Ephexin4 in cell division as previously reported (3, 4). Cells (HeLa S3/WT) capable of the Dox-inducible expression of wild-type (WT) Ephexin4 (Fig. 1*C*) were synchronized with the CDK1 inhibitor RO-3306 and subjected to time-lapse imaging analysis. The control knockdown cells normally underwent cell division; however, the Ephexin4 knockdown cells exhibited a prolonged duration of cell division and misaligned chromosomes (Fig. 1*D*). The M-phase cells were classified into three groups: prophase/prometaphase (P/PM), metaphase (M), and anaphase/telophase (A/T). Typical images of each mitotic subphase are presented in Fig. S1*A*. In Fig. S1*B*, the duration of each subphase of the M phase is shown along with cell death and the misaligned chromosomes. The ratio of each category of M-phase cells at each time point shows that the duration of the prophase/prometaphase (P/PM) did not change, whereas the duration of the M phase (mitotic index), particularly metaphase (M), was prolonged following Ephexin4 knockdown (Fig. 1*E*). Due to the metaphase extension, the peak of the percentage of anaphase/telophase (A/T) cells was low and shifted rightward. Furthermore, the number of cells exhibiting chromosome misalignment was significantly increased (Figs. 1*F* and S1*B*). Re-expression of WT Ephexin4 (siEphexin4+Dox) alleviated the delay in M-phase progression (Figs. 1*E* and S1*B*). The graphs for mitotic index and metaphase ratio show almost the same trend as the control cells (siCtrl) (Fig. 1*E*). Ephexin4 knockdown–mediated chromosome misalignment was also prevented upon its re-expression (Fig. 1*F* and S1*B*), thus ruling out the off-target effects of siRNA.

Ephexin4 knockdown was also performed in A549 cells by siRNA targeting another region (siRNA #2), and the M-phase progression was examined using RO-3306. M-phase cells were classified into two groups: before (P/PM/M) and after (A/T/Cyt) the onset of anaphase. The percentage of cells that had segregated chromosomes (A/T/Cyt) decreased in the Ephexin4 knockdown (Fig. 1, *G* and *H*; siEphexin4 #2) compared with control cells (siCtrl). There was no difference in the mitotic index, indicating no effect of Ephexin4 knockdown on cell cycle progression using this experimental protocol (Fig. 1*I*). Notably, the ratio of cells exhibiting BubR1 localization at the kinetochore, especially on the misaligned chromosomes, increased in the Ephexin4 knockdown (Fig. 1, *J* and *K*), suggesting that the activation of the spindle assembly checkpoint contributes to the Ephexin4 knockdown–caused delay in the M-phase progression. These results suggest that Ephexin4 plays a role in M-phase progression by promoting chromosome alignment.

### Ephexin4 is phosphorylated at Ser41 in M phase

**Figure 2:**
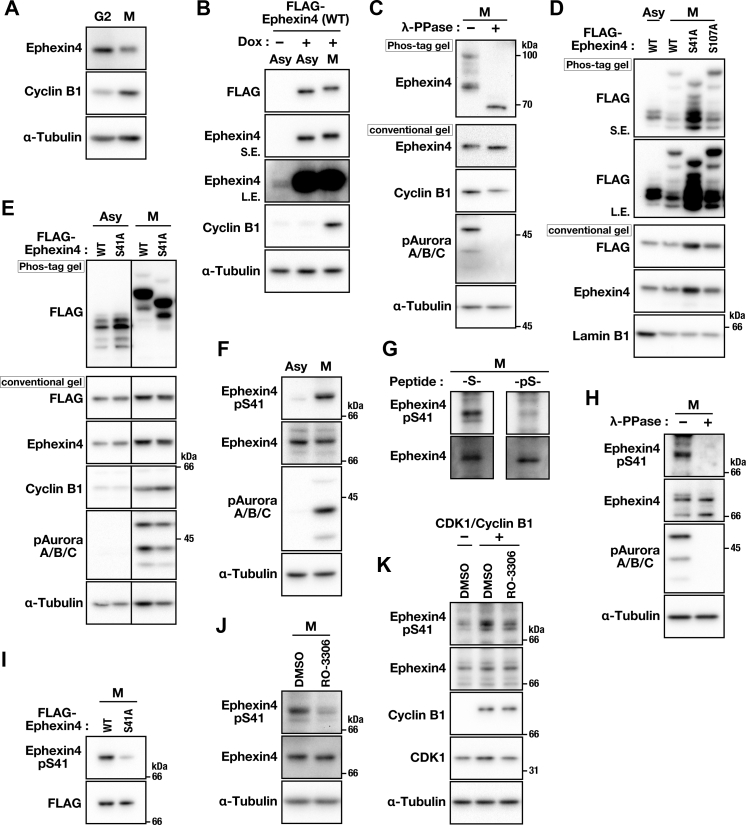
**Phosphorylation of Ephexin4 at Ser41 in M-phase.***A*, HeLa S3 cells were treated with 6 μM RO-3306 for 20 h (G2) and released for 30 min without RO-3306. After a 60-min incubation with 40 μM MG132, M-phase cells were collected by mitotic shake-off, and whole-cell lysates were subjected to Western blot (WB) analysis. *B*, A549/WT cells were treated with 1 μg/ml Dox for 48 h (Asy). To collect M-phase cells, the cells were treated with 5 μM STLC during the last 16 h of Dox treatment (M). *C*, lysates of M-phase HeLa S3 cells synchronized by a 16-h STLC treatment were incubated with Lambda protein phosphatase (λ-PPase, 400 units) at 30 °C for 1 h and separated using conventional and Phos-tag SDS-PAGE. *D*, HeLa S3 cells were transfected with 0.25 μg of plasmid DNAs encoding WT, S41A, or S107A Ephexin4 and cultured for an additional 6.5 h. The cells were then treated with 1 μg/ml Dox for 40 h, and during the last 16 h, the cells were treated with DMSO or 5 μM STLC to prepare asynchronous (Asy) and M-phase (M) cell lysates, respectively. *E*, A549/WT and A549/S41A cells were treated with Dox (WT, 1 μg/ml; S41A, 1.6 μg/ml) for 48 h, and during the last 16 h, the cells were treated with DMSO (Asy) or 5 μM STLC (M). *F*, HeLa S3 cells were treated with 5 μM STLC for 16 h. Asynchronous (Asy) and M-phase (M) cell lysates were subjected to WB analysis using anti-phospho-Ephexin4 (Ser41). *G*, M-Phase cell lysates of HeLa S3 cells synchronized with STLC were subjected to WB analysis by using anti-pSer41 antibody that was pretreated with either phosphorylated (-pS-) or unphosphorylated (-S-) epitope peptides. *H* and *I*, WB analysis was performed using the lysates of *C* (*H*) and *D* (*I*). *J*, HeLa S3 cells were treated with 5 μM STLC for 16 h and during the last 3 h, the cells were simultaneously treated with 40 μM MG132 to prevent mitotic exit. During the last 30 min of STLC/MG132 treatment, the cells were further treated with either DMSO or 6 μM RO-3306. The M-phase cells were collected by mitotic shake-off and subjected to WB analysis. *K*, HeLa S3 cells were transfected with 0.125 μg of plasmid DNAs encoding constitutively active CDK1 (CDK1 T14A/Y15F mutation) and MmGFP-fused nondegradable cyclin B1 (cyclin B1 R42A mutation) and cultured for 24 h. The cells were treated with DMSO or 20 μM RO-3306 for 2 h.

Western blot analysis revealed that endogenous (Fig. 2*A*) and FLAG-tagged (Fig. 2*B*) Ephexin4 in M-phase migrated only slightly slower compared with that obtained from RO-3306–treated G2 and asynchronous cells (Asy), respectively, suggesting that Ephexin4 is modified posttranslationally in M phase. Phos-tag SDS-PAGE, in which phosphorylated proteins are trapped by a Phos-tag and migrate slower compared with unphosphorylated proteins, indicated that Ephexin4 from M-phase cells migrated as multiple bands without Lambda phosphatase treatment, while the treatment resulted in a fast-migrating single band (Fig. 2*C*), suggesting that Ephexin4 may be phosphorylated at multiple amino acid residues. The phosphorylation of Aurora A and B kinases was also lost following phosphatase treatment (Fig. 2*C*), which validates phosphatase activity. To identify the phosphorylated amino acid residues, two Ser residues at 41 (S41A) and 107 (S107A) were independently substituted with Ala. The Phos-tag gel electrophoresis revealed an altered band pattern for S41A but not S107A from WT Ephexin4 in M-phase cells (Fig. 2, *D* and *E*), suggesting that Ser41 may be mainly phosphorylated in M phase. Ephexin4 in interphase cells also displayed a migrating pattern with multiple bands on the Phos-tag gel, regardless of the serum stimulation of the starved cells, differing from those observed in M-phase cells (Fig. 2, *D* and *E*, S2*A*), which suggests that Ephexin4 may be regulated by phosphorylation in both M-phase and interphase in distinct ways. We generated a specific antibody against phosphorylated Ephexin4 at Ser41. The antibody detected a clear band in the lysate from M-phase cells but only a faint band in the lysate from asynchronous cells (Fig. 2*F*). This band was also observed in nontransformed telomerase-immortalized hTERT RPE-1 cells upon synchronization at M phase; however, it was not detected when serum-starved quiescent hTERT RPE-1 cells were stimulated by 10% serum (Fig. S2*B*). The immunoreactivity of this antibody was lost by preincubating it with phosphorylated epitope peptides (Fig. 2*G*), a lambda phosphatase treatment of lysates (Fig. 2*H*), and the substitution of Ser41 to Ala in Ephexin4 (Fig. 2*I*), validating its specificity against Ephexin4 phosphorylated at Ser41. These results suggest that Ser41 phosphorylation occurs primarily in M phase. Treating M-phase cells with the CDK1 inhibitor RO-3306, but not the MEK inhibitor U0126, almost caused the loss of this phosphorylation (Fig. 2*J*, S2*C*). When cells were transfected with constitutively active CDK1 and nondegradable cyclin B1 (3, 15), phosphorylation at Ser41 was increased and suppressed upon simultaneous treatment with RO-3306 (Fig. 2*K*). Taken together, these results suggest that Ephexin4 is phosphorylated at Ser41 downstream of CDK1 in M phase.

### Involvement of Ser41 phosphorylation in cell division

**Figure 3:**
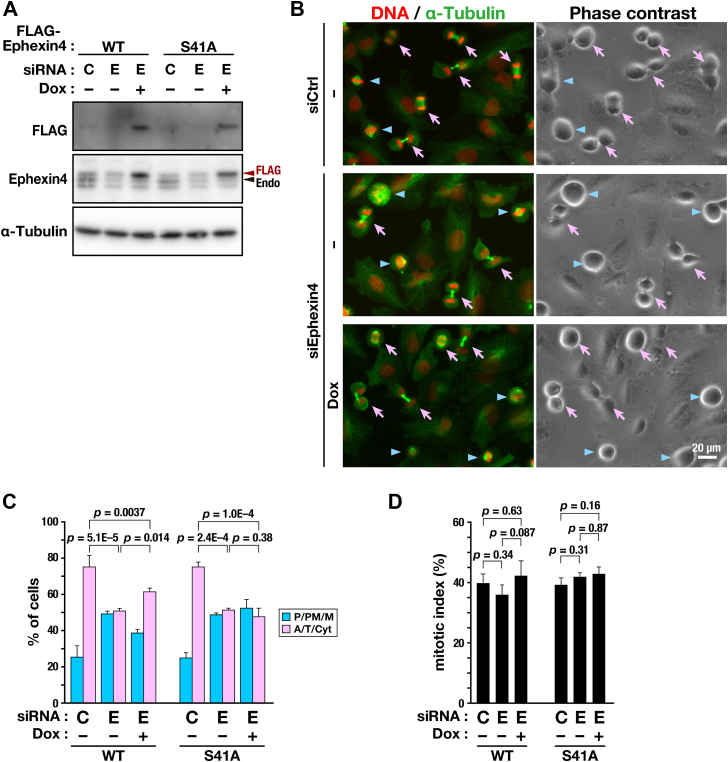
**Inhibition of Ephexin4 phosphorylation delays M-phase progression.***A*, A549/WT and A549/S41A were transfected with Ephexin4-targeting (E, #1, 1.25 nM) or nontargeting (siCtrl, 1.25 nM) siRNA and cultured for 48 h with or without 25 ng/ml Dox. Whole-cell lysates were subjected to Western blot analysis. *B*–*D*, A549/WT and A549/S41A cells were transfected with Ephexin4-targeting (E, #1, 1.25 nM) or nontargeting (C, 1.25 nM) siRNA and cultured for 48 h with or without 25 ng/ml Dox. During the last 20 h, the cells were treated with 6 μM RO-3306. The cells were then cultured for 60 min without RO-3306 and fixed with 2% formaldehyde. M-phase cells were classified into two groups: before (*blue arrowheads* in *B*; P/PM/M in *C*) and after (*pink arrows* in *B*; A/T/Cyt in *C*) the onset of anaphase based on DNA and microtubule morphologies. Representative images of A549/WT cells are shown in *B*. Scale bar represents 20 μm. The percentage of each group (*C*, WT, n ≥ 341 cells; S41A, n ≥ 393 cells per condition) and the mitotic index (*D*, WT, n ≥ 1021 cells; S41A, n ≥ 1009 cells per condition) are shown as the mean ± SD of three or four independent experiments. Tukey’s test was used to determine *p*-values.

To determine the role of phosphorylation of Ephexin4 at Ser41 during cell division, endogenous Ephexin4 was knocked down, and the effects of re-expression of the WT or S41A mutant on M-phase progression were evaluated (Fig. 3*A*). Similar to the results in Figure 1, Ephexin4 knockdown in A549/WT cells delayed M-phase progression (Fig. 3, *B* and *C*), and re-expression of WT Ephexin4 alleviated the delay caused by Ephexin4 knockdown (WT, E, and Dox+). Conversely, the Ephexin4 knockdown–mediated delay in chromosome segregation of A549/S41A cells was not prevented by re-expression of the Ephexin4-S41A mutant (S41A, E and Dox+). No significant difference in mitotic index was observed among the conditions (Fig. 3*D*), which suggests no effect on cell cycle progression. These results suggest that phosphorylation of Ephexin4 at Ser41 is required for proper M-phase progression.

**Figure 4:**
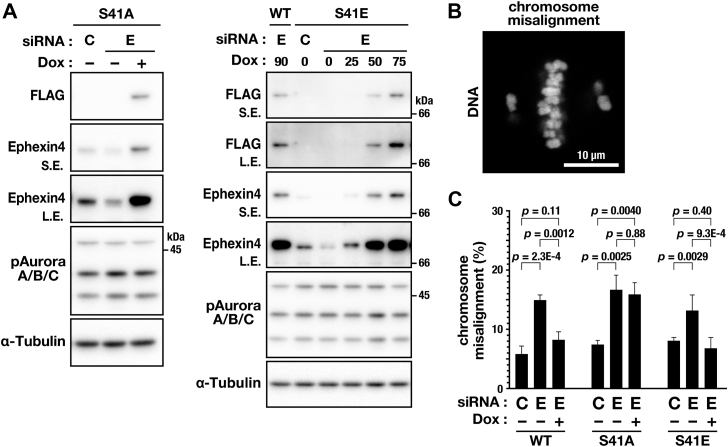
**Inhibition of Ephexin4 phosphorylation causes chromosome misalignment.***A*, HeLa S3/S41A, HeLa S3/WT, and HeLa S3/S41E cells were transfected with Ephexin4-targeting (E, #3, 250 nM) or nontargeting (C, 250 nM) siRNAs. After 7.5 h of transfection, the cells were treated with 4 mM thymidine and Dox (WT, 90 ng/ml; S41A, 75 ng/ml; S41E, 0, 25, 50, 75 ng/ml) for 24 h. After a 6-h release from thymidine, the cells were treated with 6 μM RO-3306 for 10.5 h. After a 30-min release from RO-3306, the cells were treated with 40 μM MG132 for 90 min. M-phase cells were collected by mitotic shake-off, and whole-cell lysates were subjected to Western blot analysis. For Dox-inducible expression, cells were continuously treated with Dox from the siRNA transfection until end of RO-3306 treatment. *B* and *C*, HeLa S3/WT, HeLa S3/S41A, and HeLa S3/S41E were transfected with Ephexin4-targeting (E, #3, 250 nM) or nontargeting (C, 250 nM) siRNA and cultured for 48 h with or without Dox (WT, 90 ng/ml; S41A, 75 ng/ml; S41E, 50 ng/ml). During the last 20 h, the cells were treated with 6 μM RO-3306. After a 30-min release from RO-3306, the cells were treated with 40 μM MG132 for 90 min and fixed for DNA staining. Representative images of misaligned chromosomes are shown (*B*). The percentage of cells exhibiting chromosome misalignment is shown as the mean ± SD of three independent experiments (WT, n ≥ 143 cells; S41A, n ≥ 203 cells; S41E, n ≥ 173 cells per condition) (*C*). Tukey’s test was used to determine *p*-values. Scale bar represents 10 μm.

Because phosphorylation of Ephexin4 at Ser41 is required for proper M-phase progression (Fig. 3), the requirement for chromosome alignment was determined. In metaphase cells synchronized by MG132 treatment after mitotic entry, Ephexin4 knockdown increased the ratio of cells with misaligned chromosomes compared with control siRNA (Figs. 4, S3, *A*–*C*). Re-expression of WT and S41E Ephexin4 reduced the ratio of cells with misaligned chromosomes, whereas the S41A mutant did not (Fig. 4*C* and S3*C*). These results suggest that Ephexin4 phosphorylation at Ser41 is required for chromosome alignment at the mitotic spindle equator during metaphase.

### Ser41 phosphorylation promotes RhoG localization at the plasma membrane *via* RhoG activation

**Figure 5:**
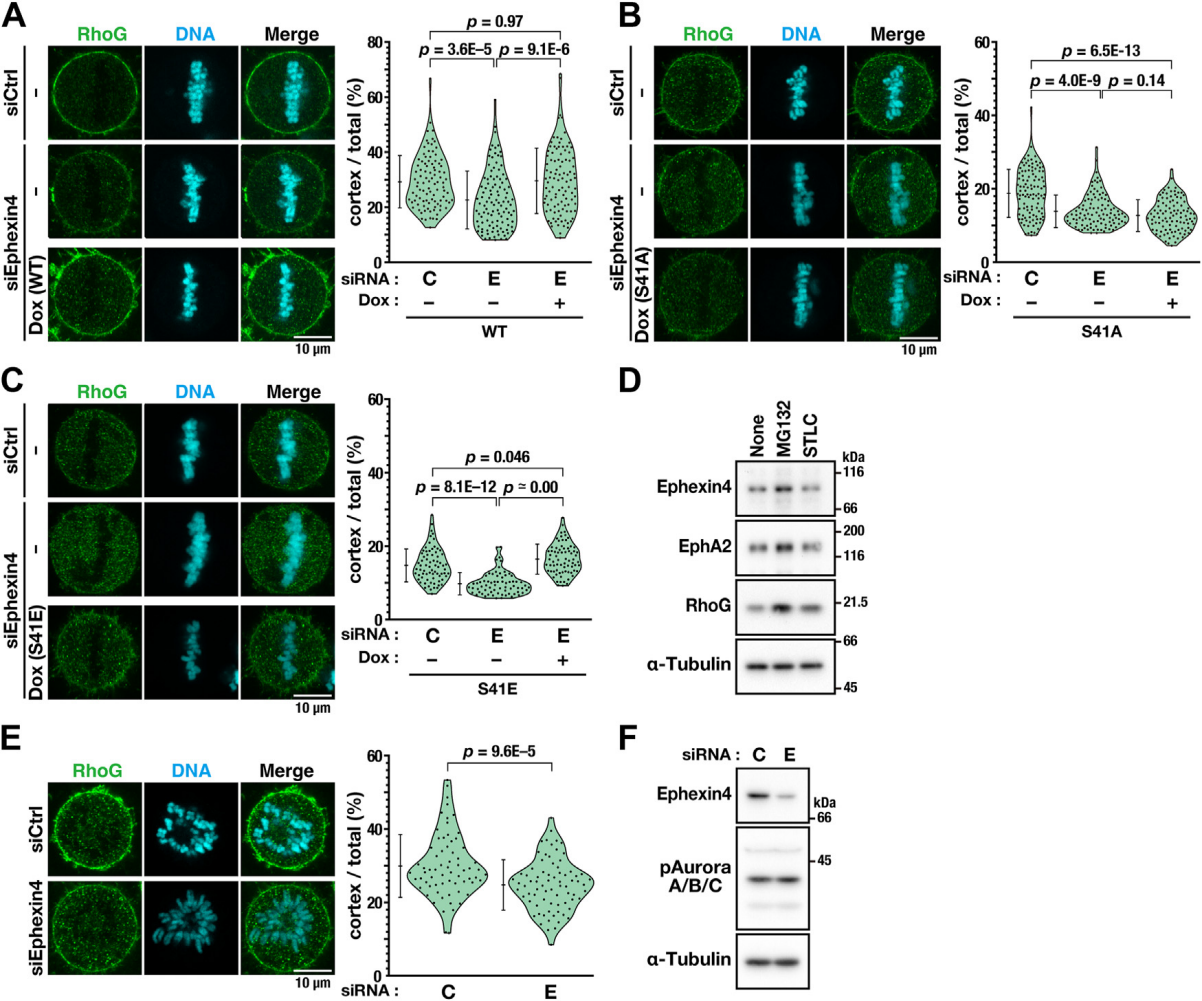
**Plasma membrane localization of RhoG requires Ephexin4 phosphorylation.***A*–*C*, HeLa S3/WT, HeLa S3/S41A, and HeLa S3/S41E cells were treated with Ephexin4-targeting (E, #3, 250 nM) or nontargeting (C, 250 nM) siRNA for 48 h with or without Dox (*A*, WT, 90 ng/ml; *B*, S41A, 75 ng/ml; *C*, S41E, 50 ng/ml). During the last 20 h, the cells were treated with 6 μM RO-3306. After a 30-min release from RO-3306, the cells were treated with 40 μM MG132 for 90 min and fixed with 4% formaldehyde. The fluorescence intensity of RhoG at the plasma membrane and that in the whole-cell area were quantified in metaphase cells. The ratio of RhoG at the plasma membrane *versus* the whole-cell area is plotted as the mean ± SD of three (*A*, *B*) or two (*C*) independent experiments (*A*, n ≥ 31 cells; *B*, n ≥ 31 cells; *C*, n ≥ 33 cells per condition). Tukey’s test (*A*) or Games-Howell test (*B*, *C*) was used to determine *p*-values. Scale bars represents 10 μm. *D*, HeLa S3 cells were treated with 6 μM RO-3306 for 20 h. After a 30-min release from RO-3306, whole-cell lysates of M-phase cells were subjected to Western blot analysis. When treated with 40 μM MG132 or 5 μM STLC, cells were incubated with these agents for 90 min before cell harvest. *E* and *F*, HeLa S3 cells were treated with Ephexin4-targeting (E, #3, 250 nM) or nontargeting (C, 250 nM) siRNA for 48 h. During the last 16 h, cells were treated with STLC and fixed. RhoG localization was analyzed with as shown in A (*E*, sum of two independent experiments, n ≥ 68 cells per condition), and whole-cell lysates of M-phase cells were subjected to Western blot analysis (*F*).

We previously reported that Ephexin4 knockdown causes a loss of RhoG localization from the plasma membrane (3). Therefore, we determined whether Ephexin4 phosphorylation at Ser41 is involved in RhoG localization at the plasma membrane. As previously reported (3), RhoG was localized at the plasma membrane in control cells and Ephexin4 knockdown reduced the RhoG localization levels (Fig. 5*A*). The RhoG localization was restored following re-expression of WT Ephexin4 to levels comparable with that of the control cells (Figs. 5*A*, S3*D*) without affecting RhoG protein levels (Fig. S3*E*). RhoG localization at the plasma membrane in Ephexin4 knockdown cells was restored by re-expression not by S41A (Fig. 5*B*), but rather by S41E Ephexin4 (Fig. 5*C*). Cells were synchronized in metaphase by treatment with MG132, which slightly increased the protein expression levels of Ephexin4, EphA2, and RhoG (Fig. 5*D*). Notably, a reduced RhoG localization was observed upon Ephexin4 knockdown, even when cells were synchronized with S-trityl-L-cysteine (STLC) without MG132 (Fig. 5, *E* and *F*), indicating that a slight increase in these proteins' expression levels induced by MG132 did not affect the phenotype observed upon Ephexin4 knockdown. Taken together, these results suggest that Ephexin4 phosphorylation at Ser41 is required for the RhoG localization at the plasma membrane.

**Figure 6:**
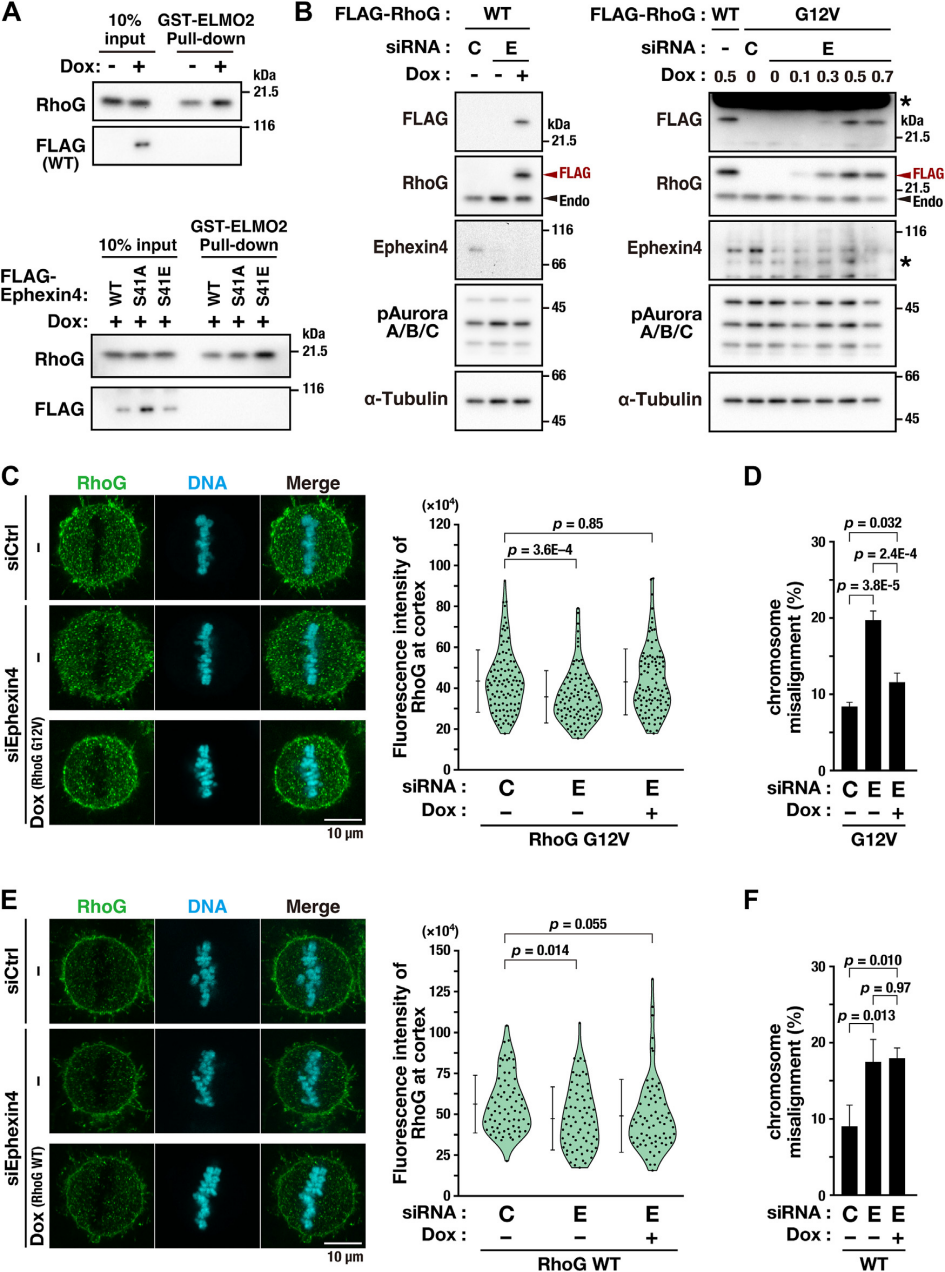
**Active RhoG-G12V is localized to the plasma membrane.***A*, expression of WT and mutant Ephexin4 were induced by treating HeLa S3/WT, HeLa S3/S41A, and HeLa S3/S41E cells with Dox, and their cell lysates were incubated with glutathione beads precoated with the N-terminal region of ELMO2. The pull down samples were analyzed by Western blot analysis. *B*, HeLa S3/RhoG WT and HeLa S3/RhoG G12V cells were transfected with Ephexin4-targeting (E, #3, 250 nM) or nontargeting (C, 250 nM) siRNA. After 7.5 h of transfection, the cells were treated with 4 mM thymidine with or without Dox (WT, 0.5 μg/ml; G12V, 0, 0.1, 0.3, 0.5, 0.7 μg/ml) for 24 h. After a 6-h release from thymidine, the cells were treated with 6 μM RO-3306 for 10.5 h, followed by release from RO-3306 for 30 min. The cells were then treated with 40 μM MG132 for 90 min, and whole-cell lysates of M-phase cells collected by mitotic shake-off were subjected to Western blot analysis. *C*–*F*, HeLa S3/RhoG WT and HeLa S3/RhoG G12V cells were transfected with Ephexin4-targeting (E, #3, 250 nM) or nontargeting (C, 250 nM) siRNA. After 7.5 h of transfection, the cells were treated with or without 0.5 μg/ml Dox for 20.5 h. Then, the cells were treated with 6 μM RO-3306 and continuously with Dox for 20 h. After a 30-min release from RO-3306, the cells were treated with 40 μM MG132 for 90 min and then fixed for DNA and RhoG staining. The fluorescence intensity of RhoG at the plasma membrane was quantified in metaphase cells and is plotted as the mean ± SD of three (*C*) or two (*E*) independent experiments (*B*, n ≥ 34 cells; *D*, n ≥ 35 cells per condition). Dunnett test was used to determine *p*-values. Scale bars represent 10 μm (*C*, *E*). The percentage of cells exhibiting chromosome misalignment is plotted as the mean ± SD of three independent experiments (*D*, n ≥ 172 cells; *F*, n ≥ 169 cells per condition). Tukey’s test was used to determine *p*-values (*D*, *F*).

Because Ephexin4 is a RhoG GEF, we next analyzed whether Ephexin4 phosphorylation at Ser41 affected RhoG activation. The N-terminal region of ELMO2 (aa1-362), which binds active RhoG (16), was fused with glutathione S-transferase (GST), and a pulldown experiment was performed. The inducible expression of WT Ephexin4 increased RhoG-GTP level (Fig. 6*A*). Under these experimental conditions, the S41E mutant further increased RhoG-GTP level in interphase. Given that Ser41 is not phosphorylated in interphase, these results suggest that Ser41 phosphorylation may enhance Ephexin4 GEF activity in M phase. Based on the result that Ephexin4 knockdown reduced RhoG localization at the plasma membrane (Fig. 5), we hypothesized that RhoG would need to be activated to localize to the plasma membrane. As expected, although endogenous RhoG localization was reduced at the plasma membrane upon Ephexin4 knockdown, exogenously expressed, active RhoG-G12V (Fig. 6, *B* and *C*) was localized to the plasma membrane even in Ephexin4-knockdown cells. Moreover, chromosome misalignment increased by Ephexin4 knockdown was prevented by the expression of RhoG-G12V (Fig. 6*D*). In sharp contrast, in Ephexin4-knockdown cells, wild-type RhoG was not localized to the plasma membrane and did not prevent the chromosome misalignment (Fig. 6, *E* and *F*). These results suggest that active RhoG is localized to the plasma membrane and that Ephexin4-pSer41–active RhoG axis has an important role in chromosome alignment.

### Inhibition of S41 phosphorylation increases vincristine sensitivity

**Figure 7:**
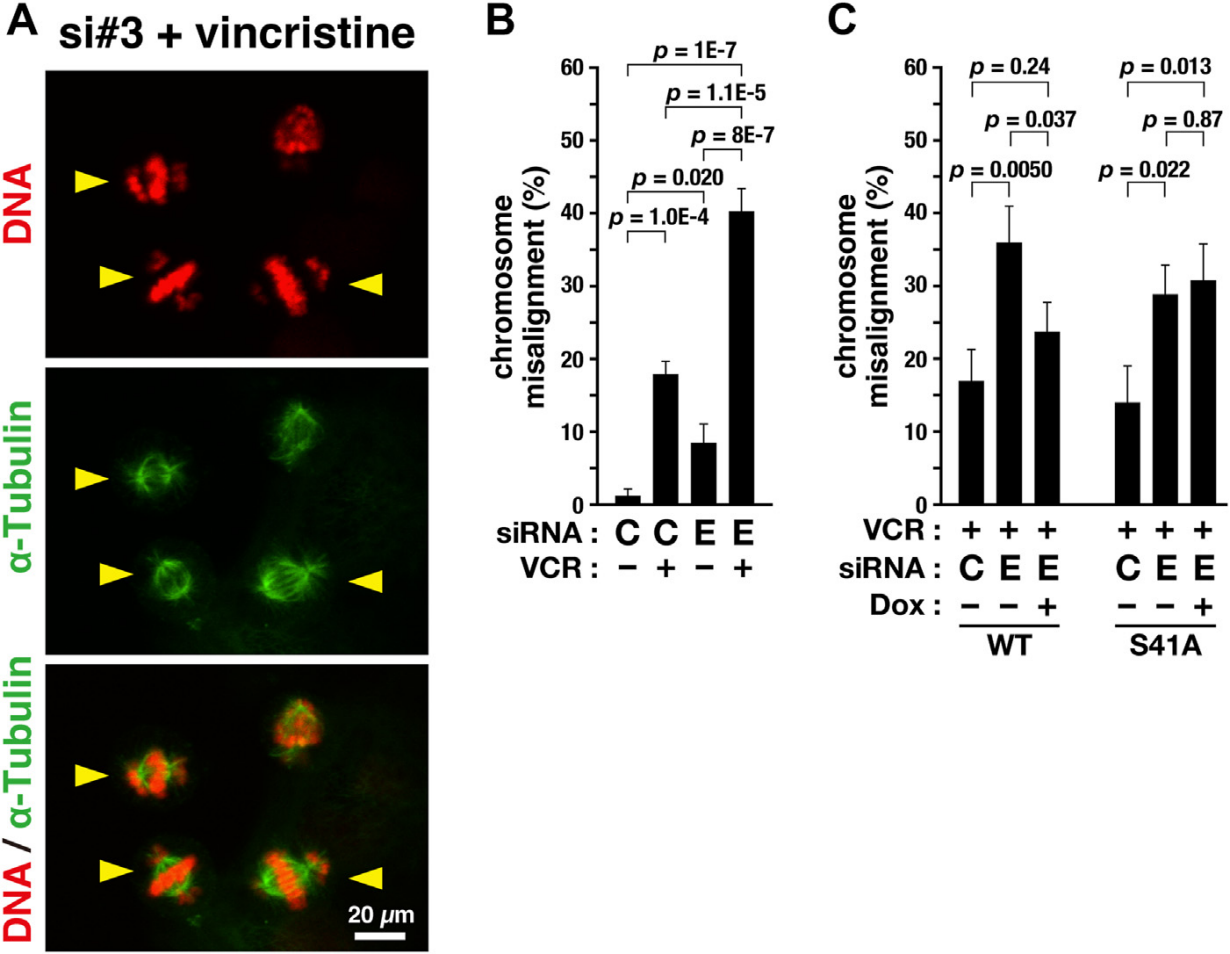
**Ephexin4 knockdown exacerbates vincristine-induced chromosome misalignment.***A* and *B*, HeLa S3 cells were transfected with Ephexin4-targeting (E, #3, 100 nM) or nontargeting (C, 100 nM) siRNA and cultured for 48 h. During the last 16 h, the cells were treated with 1.5 nM vincristine (VCR). The cells were fixed and stained for DNA and α-tubulin. Representative images of cells exhibiting chromosome misalignment (*arrowheads*) are shown. Scale bar represents 10 μm (*A*). The percentage of cells exhibiting chromosome misalignment is shown as the mean ± SD calculated from three independent experiments. (*B*, n ≥ 103 cells per condition). Tukey’s test was used to determine *p*-values. *C*, HeLa S3/WT and HeLa S3/S41A cells were transfected with Ephexin4-targeting (E, #3, 250 nM) or nontargeting (C, 250 nM) siRNA and cultured for 48 h with or without Dox (WT, 90 ng/ml; S41A, 75 ng/ml). During the last 16 h, the cells were treated with 1.5 nM VCR. The percentage of cells with misaligned chromosomes is shown as the mean ± SD calculated from three independent experiments (n ≥ 163 cells per condition). Tukey’s test was used to determine *p*-values.

Microtubule-targeting anticancer agents, including paclitaxel and vincristine (VCR), generate severe M-phase defects, thereby causing cell death; these agents have been routinely used for cancer treatment. Because the inhibition of S41 phosphorylation caused chromosome misalignment, we examined whether the inhibition of Ephexin4 phosphorylation enhanced the effects of VCR. HeLa S3 cells were transfected with a nontargeting control (C) or Ephexin4-targeting (E, #3) siRNA. After 32 h, the cells were treated with 1.5 nM VCR for 16 h (Fig. 7, *A* and *B*). While only 1.3% of the control siRNA-treated cells without VCR exhibited chromosome misalignment, treatment with 1.5 nM VCR or siEphexin4 (E, #3) resulted in 17.8% or 8.5% of the cells exhibiting chromosome misalignment, respectively. Interestingly, 40% of the cells showed chromosome misalignment upon simultaneous treatment with VCR and siEphexin4 (Fig. 7*A*, yellow arrowheads, and *B*), suggesting that the inhibition of Ephexin4 potentiates the effect of VCR. This potentiation was also observed in HeLa S3/WT and HeLa S3/S41A cells and was prevented by the re-expression of WT Ephexin4 (Fig. 7*C*). In sharp contrast, re-expression of the Ephexin4-S41A mutant did not prevent it, suggesting that the inhibition of Ephexin4 phosphorylation potentiates the effects of low-dose VCR.

### Overexpression of Ephexin4 causes abnormal cyst formation

**Figure 8:**
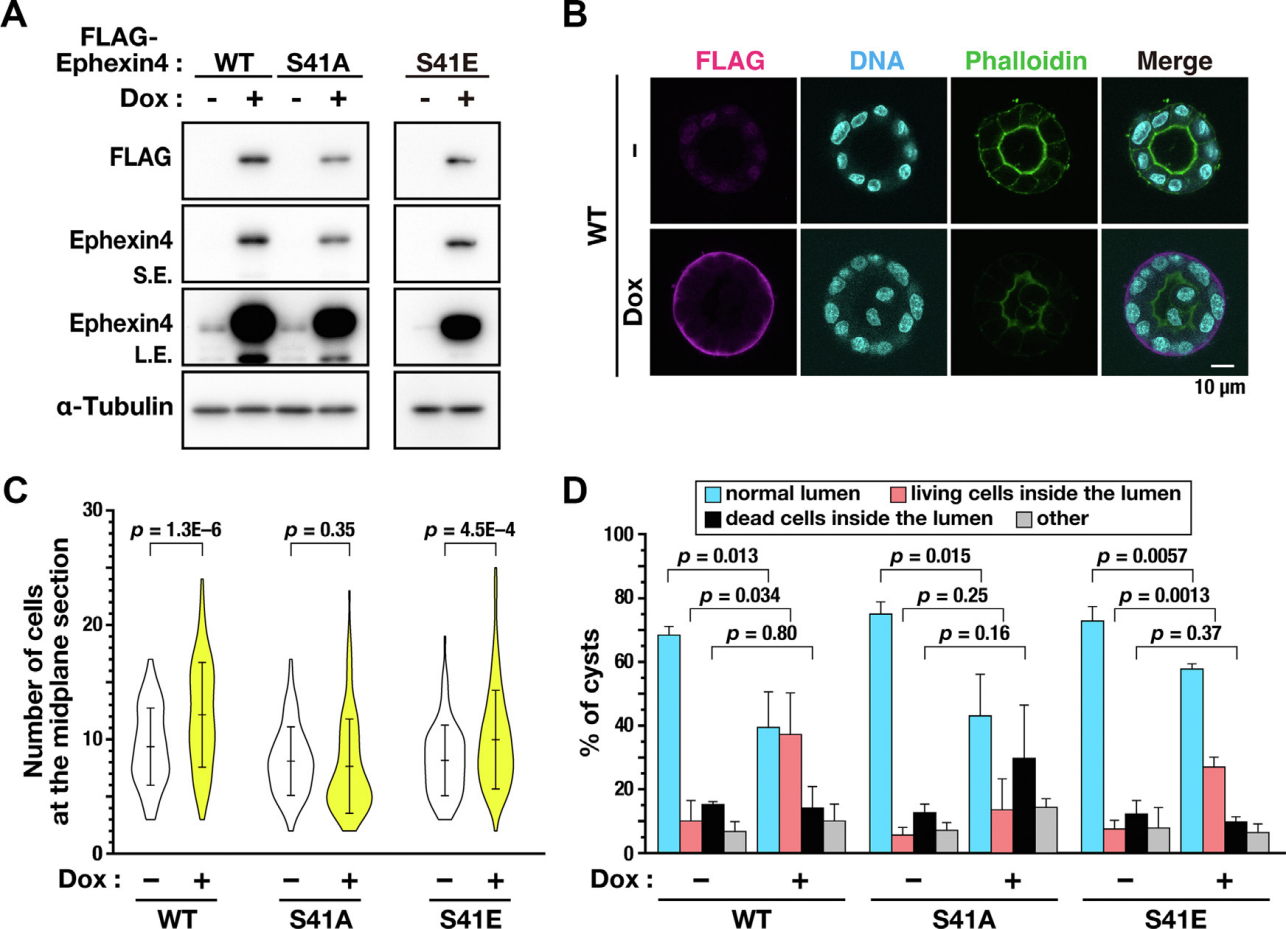
**Overexpression of Ephexin4 disrupts the epithelial morphogenesis.***A*, MDCK/WT, MDCK/S41A, and MDCK/S41E cells were cultured for 48 h with 3 μg/ml Dox. Whole cell lysates were subjected to Western blot analysis. *B*–*D*, MDCK/WT, MDCK/S41A, and MDCK/S41E cells were grown in 3D cultures with or without 3 μg/ml Dox in 2% Matrigel for 5 days. The cysts were fixed and stained, and the representative images were displayed. Scale bar represents 10 μm (*B*). The number of cells at the midplane section is plotted as the mean ± SD calculated from three independent experiments (*C*; n ≥ 63 cyst per condition). The *p*-values were determined using the Welch’s *t* test (*C*). The percentage of cysts with normal lumen, cysts with living cells, or dead cells inside single lumen is shown as the mean ± SD calculated from three independent experiments (*D*; n ≥ 63 cyst per condition). The *p*-values were determined using the Student’s or Welch’s *t* test (*D*).

Ephexin4 is overexpressed in various cancer cells (14). Because Ephexin4 plays a role in the regulation of cell division, we hypothesized that dysregulation of cell division by overexpression of Ephexin4 would cause cancer development. Most cancers arise from epithelial tissue, in which cells maintain cell polarity. Loss of cell polarity and cell-cell adhesion may be associated with the early stages of cancer and its progression. This can be modeled by cyst formation using a 3D Matrigel culture of Madin-Darby canine kidney (MDCK) cell. Because cyst formation requires proper orientation of the mitotic spindle axis in cell division based on the apical-basolateral polarity, the effects of overexpressing Ephexin4 on MDCK cyst formation and the involvement of Ephexin4 phosphorylation at Ser41 were examined. MDCK cells capable of Dox-inducible expression of WT, S41A and S41E Ephexin4 were generated (Fig. 8*A*). When the cell clusters consisted of more than five cells after a 5-day 3D Matrigel culture, approximately 68.2% of the cell clusters formed a cyst enclosing a single hollow lumen by a spherical monolayer (Fig. 8*B*). F-actin accumulation on the apical membrane indicates the formation of polarized cysts (Fig. 8*B*). Moreover, FLAG-Ephexin4 was localized on the basal membrane irrespective of S41A and S41E mutations (data not shown). Upon overexpressing the WT and S41E Ephexin4, the number of cells at the midplane section of the cysts increased compared with the Dox-untreated cells (Fig. 8*C*), suggesting an increase in cell proliferation. In addition, although apical-basolateral polarity was established, the number of cysts with cells inside the lumen was significantly increased (Fig. 8*D*, WT and S41E). On the other hand, S41A Ephexin4 mutant expression did not result in these phenotypes (Fig. 8*D*). If the spindle axis is not parallel to the apical membrane during cell division, one of the daughter cells can be extruded into the cyst lumen. Therefore, the Ephexin4 overexpression may disrupt normal cyst formation, possibly through the deregulation of cell division by overexpressed, S41-phosphorylated Ephexin4.

## Discussion

**Figure 9:**
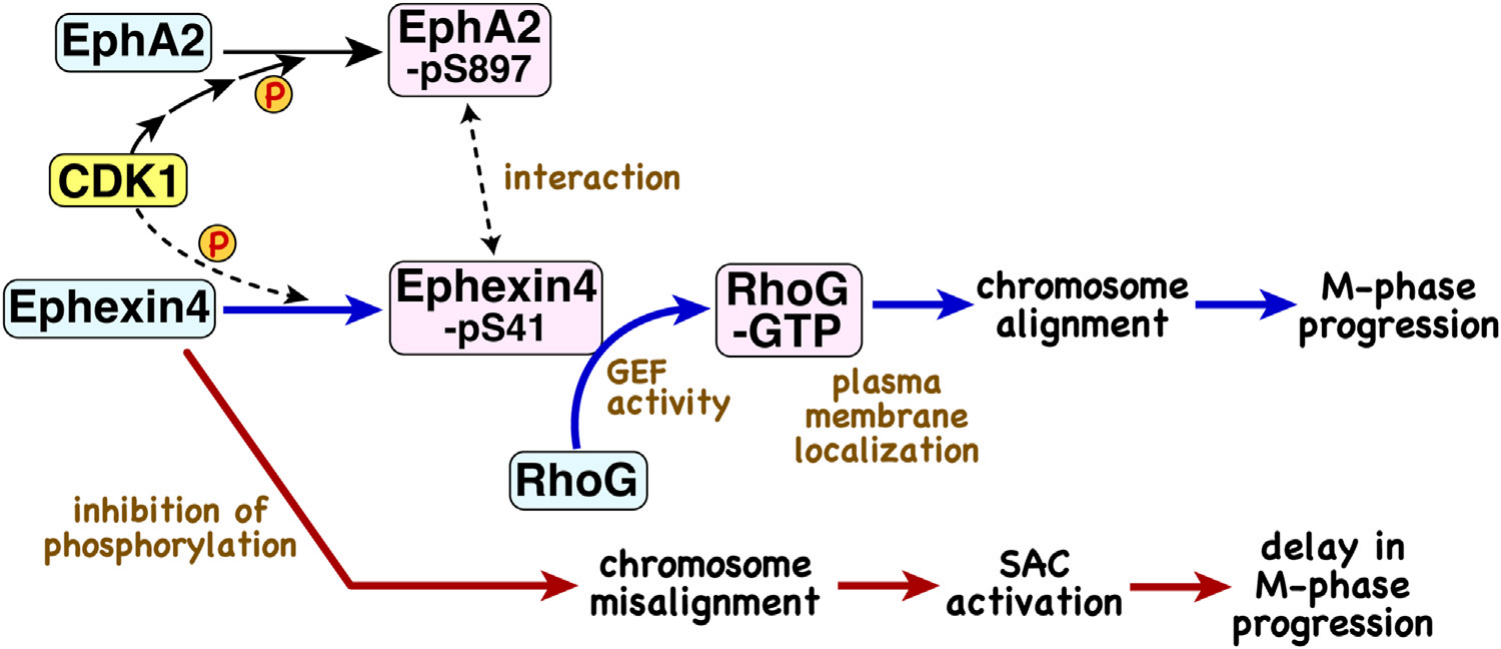
**Model of cell division regulation by the EphA2–Ephexin4–RhoG signaling pathway.** EphA2 and Ephexin4 are phosphorylated downstream of CDK1, and their interaction promotes localization of RhoG-GTP to the plasma membrane. Phosphorylated Ephexin4 at S41 activates RhoG, which promotes M-phase progression through chromosome alignment (*blue* line). Inhibition of Ephexin4 phosphorylation leads to misalignment of chromosomes, resulting in delay in M-phase progression *via* SAC activation (*red* line).

In this study, we demonstrated that Ephexin4 is phosphorylated at Ser41, and this phosphorylation event is necessary for accurate chromosomal alignment. The chromosome misalignment caused by Ephexin4 knockdown was suppressed by the expression of a constitutive active RhoG-G12V mutant. Ephexin4–RhoG axis may contribute to chromosome alignment. A signal model summarizing this study and our previous results (3) is displayed in Figure 9. This is the first report that demonstrates the involvement of phosphorylation in regulating Ephexin4 in M phase. Ephexin4 is overexpressed in some cancer types (1, 14), and activation of RhoG promotes cancer cell invasion, migration, and cell proliferation (2, 17). Therefore, our findings are important for developing novel strategies for cancer treatment.

The RhoG-GEF Ephexin4 activates RhoG in response to growth factor signaling and mediates cell migration in interphase (2, 17). We previously reported that Ephexin4 is recruited to the receptor-type tyrosine kinase EphA2, which is phosphorylated downstream of the CDK1–MEK–ERK–RSK signaling pathway at Ser897 in M phase. This results in the plasma membrane localization of RhoG (3). Inhibition of this pathway causes M-phase delay, accompanied by a reduction of RhoG localization at the plasma membrane. Low concentrations of cytochalasin B cause bleb formation, which is exacerbated by EphA2 knockdown. This suggests that the EphA2–Ephexin4–RhoG axis regulates plasma membrane rigidity in M phase through actin remodeling. Therefore, we expected the presence of M phase–specific regulatory mechanisms for Ephexin4 that were distinct from the interphase. In the present study, Phos-tag SDS-PAGE analysis revealed multiple phosphorylation sites in M phase, which were greater than interphase (Fig. 2, *C* and *D*). This supports our hypothesis that phosphorylation at multiple sites results in distinct roles for Ephexin4 in M phase compared with interphase.

The portal site for the modification of proteins by phosphorylation, PhosphositePlus (https://www.phosphosite.org/), shows several phosphorylation sites in Ephexin4. Because CDK1 is a master regulator of cell division, we focused on the amino acid sequence SPXK/R, which is a consensus site for CDK1. Among the possible multiple phosphorylation sites, Ser41 was identified as a phosphorylation site (Fig. 2, *D*–*I*). The S41A Ephexin4 mutant did not rescue Ephexin4 knockdown–mediated M-phase delay (Fig. 3), chromosome misalignment (Fig. 4), and reduction of RhoG localization to the plasma membrane (Fig. 5*B*), suggesting the requirement of Ephexin4 phosphorylation at Ser41 in cell division. In contrast to the S41A mutant, the phosphomimetic S41E mutant rescued the Ephexin4 knockdown–mediated chromosome misalignment (Fig. 4) and restored the plasma membrane localization of RhoG with a slight increase in RhoG localization (Fig. 5*C*) with increased RhoG activation level (Fig. 6*A*), supporting a requirement of Ephexin4 phosphorylation. This phosphorylation was detected by this antibody in M phase but not in interphase (Fig. 2*F*) and was suppressed by the CDK1 inhibitor (Fig. 2, *I* and *J*) but not the MEK inhibitor (Fig. S2*C*). These results indicate that Ephexin4 phosphorylation at Ser41 occurs in M phase downstream of CDK1 and plays a crucial role in M phase, although the kinase directly phosphorylating Ephexin4 at S41 remains unknown.

In addition to Tyr phosphorylation for the regulation of GEF activity in the Dbl protein family for Rho GTPases, Ser/Thr phosphorylation of GEF has been reported. The RhoA GEF, GEF-H1, is activated by ERK1/2-mediated phosphorylation (18). In contrast, phosphorylation by Aurora A/B and CDK1 inhibits GEF-H1 activity (19). Tiam1 is phosphorylated by Ca^2+^/calmodulin-dependent protein kinase II, thus enhancing its GEF activity toward Rac1 (20). The present study is the first to demonstrate that the GEF activity of Ephexin4 is regulated by Ser/Thr phosphorylation in M phase. What is the mechanism underlying Ephexin4 activation by Ser41 phosphorylation? Members of the Dbl protein family contain the DH domain and adjacent PH domain. Dbl proteins are often activated by tyrosine phosphorylation, which relieves them from autoinhibition. Tyrosine phosphorylation of Vav at Tyr174 by the Src family tyrosine kinase Lck activates its GEF activity (21). Truncation of the N-terminal region containing this Src family kinase–recognition site results in Vav-2 activation, which is independent of phosphorylation (22). Furthermore, NMR spectroscopy revealed that phosphorylation at this site releases the N-terminal extension away from the DH domain, relieving autoinhibition and exposing the GEF active site of the DH domain (23).

Similar to Vav, autoinhibition regulates the GEF activity of the Ephexin protein subfamily. Ephexin proteins have a tandem DH-PH domain, followed by an Src homology 3 (SH3) domain, except for Ephexin5 (1). Phosphorylation of the Tyr residue of the Ephexin subfamily proteins (Ephexin1–5) plays an important role in regulating their GEF activities. Ephexin proteins contain a conserved LYQ sequence in the helix [referred to as an inhibitory helix in (9)] in the N-terminal region (9, 12). Because of the intramolecular interaction between the inhibitory helix and the DH domain, Ephexins adopt an inhibitory conformation (9). In addition, the C-terminal region containing an SH3 domain is involved in autoinhibition of the Ephexin proteins (8, 9, 10, 11). Interaction of the SH3 domain with the polyproline region promotes the interaction of the inhibitory helix and the DH domain. The latter interaction is destabilized through binding of the SH3 domain with another protein (8). In addition to disruption of the interaction between the SH3 and the polyproline region, phosphorylation of the Tyr residue in the inhibitory helix relieves the intramolecular inhibitory interaction between the inhibitory helix and the DH domain, thus activating GEF activity (7, 8, 9, 12). Src is responsible for this phosphorylation in Ephexin1–3 (8).

For Ephexin4, complete autoinhibition is achieved through inhibitory helix- and SH3-mediated intramolecular interactions (9). In contrast to Ephexin1–3, tyrosine phosphorylation of the LYQ motif of Ephexin4 by Src has not been observed (13, 24). Phosphorylation of Ephexin4 by Fyn was reported; however, phosphorylated amino acid residues were not identified (24). Nonetheless, intramolecular interactions may be a general mechanism for the regulation of GEF activities in Ephexins. Moreover, phosphorylation may be important to break the autoinhibitory interaction in Ephexins. Consistently, we observed Ephexin4 phosphorylation in M phase and identified Ser41 as a phosphorylated residue. Although further studies are required, phosphorylation of Ephexin4 at Ser41 may relieve the inhibitory intramolecular interaction between the inhibitory helix and DH domain.

Phos-tag SDS-PAGE revealed several mobility-shifted bands of Ephexin4 in M-phase cell lysates compared with the asynchronous cell lysate (Fig. 2*D*). Substitution of Ser41 with Ala only partially abolished the mobility shift and several bands remained shifted (Fig. 2*D*), suggesting that not only Ser41 but other amino acid residues may be phosphorylated in M phase. As described above, full activation of Ephexin4 requires relief from both N- and C-terminal region–mediated inhibition. N-terminus–mediated inhibition may be relieved by Ser41 phosphorylation, and the other autoinhibitory mechanisms may be relieved by the phosphorylation of unidentified residue(s). Because Ephexin4 interacts with phosphorylated EphA2 at Ser897 to activate GEF activity, the interaction with phosphorylated EphA2 may contribute to relieving the C-terminus–mediated inhibition or facilitate the phosphorylation at Ser41. The Ephexin4 activity may be subject to multiple regulation levels in M phase.

Cyst formation is a model that has been used to study the regeneration of cellular events during epithelial morphogenesis with apical-basolateral polarity. We generated MDCK cells expressing WT, S41A, or S41E Ephexin4 (Fig. 8*A*). Cells overexpressing WT Ephexin4 generated cysts with living cells in a hollow lumen (Fig. 8*B*). Conversely, this was not observed for the S41A Ephexin4 mutant–expressing cells. Generally, the mitotic spindle axis aligns parallel to the apical membrane during cyst formation. When the spindle axis is oriented far from parallel, one of the daughter cells is extruded into the lumen. The number of cysts with cells in the lumen was increased upon WT Ephexin4 overexpression (Fig. 8*C*), suggesting that mis-orientation of the mitotic spindle axis far from parallel to the apical membrane may occur. This was observed in Ephexin4 S41E but not S41A, suggesting that phosphorylation of Ephexin4 at Ser41 may be involved in the spindle axis orientation and that overexpressing WT and S41E Ephexin4 may deregulate spindle axis orientation. If S41 phosphorylation is required to disrupt epithelial morphogenesis, the suppression of S41 phosphorylation may prevent the generation of cells inside the hollow lumen. Generally, cells inside the hollow lumen are cleared by undergoing apoptosis because of the deprivation of the cell–matrix interaction (25, 26). Because RhoG activates phosphatidyl inositol-3 kinase and, consequentially, AKT (27, 28) overexpressing WT Ephexin4 may prevent the anoikis of cells extruded into the hollow lumen. Given that the majority of cancer cells are of epithelial cell origin and Ephexin4 is overexpressed in various cancer cells, inhibition of the signaling pathway for Ser41 phosphorylation of Ephexin4 may represent a candidate target for developing novel cancer drugs.

Furthermore, when inhibition of Ser41 phosphorylation was combined with VCR, it caused severe misalignment of mitotic chromosomes (Fig. 7). In M phase, Ephexin4 participates in the regulation of plasma membrane rigidity. Cortical actin at the plasma membrane tethers the mitotic spindle *via* interaction with astral microtubules; the actomyosin cortex should be appropriately regulated to ensure mitotic spindle formation (29). Dual inhibition of membrane rigidity and microtubule polymerization may cause additional effects on mitotic spindle formation. This is supported by the finding that low-dose VCR combined with drug-targeting actin filaments cause multipolar spindles and apoptosis (30). Microtubule-targeting agents have side effects, such as peripheral neuropathy, because the β-tubulin subunit of microtubules is a critical component of nerve fiber axons (31). Therefore, a reduced concentration of these agents is desirable. The combination of Ephexin4 phosphorylation inhibition with anti-microtubule drugs may be useful for targeting cancer cells, because severe defects in chromosome segregation cause cell death. Further studies to identify the mechanisms in detail will facilitate the development of novel therapies for cancer treatment.

## Experimental procedures

### Plasmid DNA

Ephexin4 cDNA was purchased from GenScript Japan (cloneID, OHu06388). The FLAG tag and stop codon were fused at the N-terminus and the C-terminus of Ephexin4, respectively, by inverse PCR. The primer set used to introduce the FLAG tag was 5′-GACGACAAACTCGGAATGGCCCAGCGGCACTCAGAC-3′ and 5′-ATCGTCCTTGTAATCCATGGTGGAGCCTGCTTTTTTGTACAAAGTTGGC-3′. The primer set used to introduce the stop codon was 5′-TTACACGTCCGTCTCCACCCGCAG-3′ and 5′-GTAAAGCGGCCGCACTCGAGATATCTAGACC-3′. The amino acid linker sequence Leu-Gly was introduced between the FLAG and Ephexin4. The FLAG-tagged Ephexin4 was introduced into pENTR4 no ccDB to generate pENTR4/FLAG-Ephexin4. pENTR4 no ccDB (6861) was a gift from Eric Campeau & Paul Kaufman (Addgene plasmid #17424; http://n2t.net/addgene: 17424; RRID: Addgene_17424) (32).

For knockdown rescue experiments using siRNA targeting the coding region of Ephexin4 (siRNA #1, described below), a silent mutation was introduced into the target sequence by PCR using the primer set 5′-GCTCTAAAACTAGGTACCCAACAGCTGATCCCTAAGAGCC-3′ and 5′- ACCTAGTTTTAGAGCCGCCGGGCTCTCTGTG-3′. siRNA#1-resistant Ephexin4 was used as WT Ephexin4. FLAG-Ephexin4 within the pENTR4 vector was recombined into a pLIX402 lentiviral vector (a gift from David Root, Addgene plasmid #41394) *via* a Gateway LR reaction (Invitrogen) based on the manufacturer’s instructions. The Ser41Ala mutation was introduced by a PCR reaction using pENTR4/FLAG-Ephexin4 as a template and the primer set, 5′-GGCGCCCCGCGGGTTAGAGACGATGCCGCC-3′ and 5′-AACCCGCGGGGCGCCACGGACCATTGGGAG-3′. For the Ser41Glu mutation, the primer set, 5′-CGTGGCGAACCGCGTGTTAGAGACGATG-3′ and 5′-ACGCGGTTCGCCACGGACCATTGGGAG-3′ was used. For the Ser107Ala mutation, the primer set, 5′-CACCAGGCCTTCGGGGCGGCAGTACTTAGCAGGGAG-3′ and 5′-CTAAGTACTGCCGCCCCGAAGGCCTGGTGGCGGGCTG-3′ was used. The mutants were recombined into pLIX402 as described above.

The nondegradable cyclin B1 mutant fused with a modified form of GFP, (R42A)cyclin B1-MmGFP (15) and pcDNA3-cdc2-AF (plasmid 39872; Addgene) (33) were gifts from J. Pines.

To construct GST-ELMO2, the RhoG-binding domain–included N-terminal region (aa 1–362) of ELMO2 cDNA (GenScript Japan, Clone ID, OHu02300C) was amplified by PCR using the primer set, 5′-CTCGGATCCGAATTCATGCC-3′ and 5′-TTTAAGCTTCTACATGGCTGGATTGATGTGGTTGG-3′, generating DNA fragment harboring BamHI and HindIII sites. The fragment was ligated into the corresponding sites of GST-YAP2 [a gift from Kunliang Guan (Addgene plasmid #24637; http://n2t.net/addgene:24637; RRID:Addgene_24637)].

### Cell culture and transfection

Human cervix adenocarcinoma HeLa S3 cells, human lung adenocarcinoma A549 cells, and normal canine kidney MDCKn cells were obtained from the Japanese Collection of Research Biosources. Lenti-X 293T cells were obtained from Clontech Laboratories. These cells and derived cell lines capable of doxycycline (Dox)-inducible expression of WT Ephexin4 and the mutants harboring a Ser41 to Ala substitution (S41A), Ser41 to Glu substitution (S41E), or Ser107 to Ala substitution (S107A) were cultured in Dulbecco’s modified Eagle’s medium (DMEM) containing 5% fetal bovine serum and 20 mM Hepes–NaOH (pH 7.4). hTERT RPE-1 cells (CRL-4000; American Type Culture Collection) were cultured in DMEM/Ham's F-12 medium. Transfection was performed using Lipofectamine 2000 (Invitrogen) based on the manufacturer’s instructions.

### Chemicals

The reversible CDK1 inhibitor RO-3306 (S7747, Selleckchem) was used at 5 or 6 μM. The Eg5 inhibitor STLC (164739, Sigma–Aldrich) was used at 5 μM for 16 h to arrest cells in mitosis. The proteasome inhibitor MG132 (3175-v, Peptide Institute) was used at 40 μM. These chemicals were dissolved in dimethyl sulfoxide (Nacalai Tesque). Thymidine (T1895-10G, Sigma-Aldrich) was used at 4 mM. Vincristine (Nippon Kayaku) was used at 1.5 nM.

### Small interfering RNA

HeLa S3 and A549 cells were transfected with siEphexin4 #1 (1.25 nM, Hs02_00345657), siEphexin4 #2 (50 nM, Hs02_00345658), and siEphexin4 #3 (250 nM) siRNAs using Lipofectamine 2000 (Invitrogen). siEphexin4 #1 and #2 were purchased from Sigma-Aldrich, and siEphxin4 #3 was targeted to the 3′-UTR region (5′-GAGGUUUCUAAAUACAUUAUU-3′) and was synthesized by Sigma-Aldrich. As control siRNA, MISSION siRNA Universal Negative Control #1 (SIC-001, MilliporeSigma) was used.

### Establishment of stable cell lines by lentiviral transduction

To establish cell lines capable of Dox-inducible expression of FLAG-tagged WT and mutant Ephexin4, Lenti-X 293T cells were cotransfected with 1.2 μg of the Ephexin4-expressing vector and the lentiviral packaging plasmids [0.8 μg of pCMV-VSV-G-RSV-Rev and 0.8 μg of pCAG-HIVgp; gifted by Dr Hiroyuki Miyoshi (Rikagaku Kenkyusho Foundation BioResource Center)] using Lipofectamine 2000. The cells were treated with 10 μM forskolin 16 h after transfection. After 8 h, the culture medium containing virus was added to the target cells (HeLa S3, A549, and MDCK) pretreated with 80 μg/ml polybrene, and the infected cells were selected with 1 μg/ml puromycin. HeLa S3-derived HeLa S3/WT, HeLa S3/S41A, and HeLa S3/S41E, MDCK-derived MDCK/WT, MDCK/S41A, and MDCK/S41E, and A549-derived A549/WT, and A549/S41A were generated. HeLa S3 cell lines capable of Dox-inducible expression of FLAG-tagged WT RhoG and the mutant with a Gly12 to Val substitution were established as described previously (3).

### Antibodies

For immunofluorescence (IF) staining and Western blot (WB) analyses, mouse monoclonal anti-BubR1 (IF, 1:200; 8G1, K0169-3, Medical & Biological Laboratories), anti-cdc2 (CDK1) (WB, 1:1000; sc-54, Santa Cruz Biotechnology), anti-p-ERK 1/2 (WB, 1:3000; 12D4, sc-81492, Santa Cruz Biotechnology), anti-ERK 1/2 (WB, 1:2000; C-9, sc-514302, Santa Cruz Biotechnology), anti-FLAG (WB, 1:1000; M2, F1804, Sigma-Aldrich) and anti-RhoG (IF, 1:200; WB, 1:1000; 1F3 B3 E5, sc-80015, Santa Cruz Biotechnology) antibodies, rabbit monoclonal anti-phospho-Aurora A (Thr288)/Aurora B (Thr232)/Aurora C (Thr198) (WB, 1:1000; D13A11, #2914, Cell Signaling Technology), and anti-EphA2 (WB, 1:1000; 6997S, Cell Signaling Technology) antibodies, rabbit polyclonal anti-ARHGEF16 (WB, 1:1000; A301-961A, Bethyl Laboratories), anti-cyclin B1 (WB, 1:2000; H-433, sc-752, Santa Cruz Biotechnology), and anti-FLAG (IF, 1:400; WB, 1:400–1000; PM020, Medical & Biological Laboratories) antibodies, goat polyclonal anti-Lamin B1 (WB, 1:1000; C-20, sc-6216, Santa Cruz Biotechnology) antibody, rat anti-α-tubulin (IF, 1:800; WB, 1:2000; MCA78G, Bio-Rad) antibody, and human anti-centromere antigen (IF, 1:400; HCT0100, ImmunoVision) were used. Polyclonal antibody against phosphorylated Ephexin4 at Ser41 was generated (Scrum) in rabbits by immunization with phosphorylated peptide [G(pS)PRVRDDAAF] and used for WB (1:1000).

For secondary antibodies for IF analysis, Alexa Fluor 488–labeled donkey anti-mouse IgG (IF, 1:800; A21202) and donkey anti-rat IgG (IF, 1:800; A21208), AlexaFluor 555–labeled goat anti-human IgG (1:800; A21433), and Alexa Fluor 647–labeled donkey anti-rabbit IgG (IF, 1:800; A31573) antibodies were purchased from Thermo Fisher Scientific. For WB analysis, horseradish peroxidase–conjugated donkey IgG antibodies were purchased from Jackson Immuno Research. Anti-mouse (WB, 1:8000; 715-035-151), anti-rabbit (WB, 1:8000; 711-035-152), and anti-rat (WB, 1:8000; 712-035-153) IgG secondary antibodies were used. Horseradish peroxidase–conjugated bovine anti-goat IgG (WB, 1:4000; sc-2350, Santa Cruz Biotechnology) antibody was used.

### IF staining

IF staining was performed as described previously (3, 4). Briefly, cells were cultured on a coverslip and fixed with 4% formaldehyde in PBS (−) at room temperature for 20 min. When stained for BubR1 and CREST, cells were fixed with 4% formaldehyde in PBS (−) for 7 min and permeabilized with 0.2% Triton X-100 in PBS (−) for 5 min at room temperature. For blocking and permeabilization, the cells were incubated with 3% bovine serum albumin (BSA)/0.1% saponin/PBS (−) for 30 min. The cells were sequentially incubated with primary and secondary antibodies in 3% BSA/0.1% saponin/PBS (−) for 1 h each. For DNA staining, 0.5 μM Hoechst 33342 was added to the secondary antibody solution. The coverslips were mounted onto glass slides with an antifade solution.

To observe the IF images, an IX-83 fluorescence microscope (Olympus) with a 40 × 0.75 NA objective was used. A U-FUNA cube (360–370 nm excitation, 420–460 nm emission) and U-FBNA cube (470–495 nm excitation, 510–550 nm emission) were used to observe Hoechst 33342 and Alexa Fluor 488 fluorescence, respectively. The MAICO MEMS confocal unit (Hamamatsu Photonics) was used to obtain confocal fluorescence images of RhoG and BubR1 on the plasma membrane and the CREST-positive kinetochore, respectively, and FLAG, F-actin, and DNA in cyst. The z-section images of BubR1 and CREST were captured with 0.3 μm intervals and projected into a single layer by maximum intensity projection using Image J (NIH). The figures were edited using Image J, Photoshop CC (Adobe) and Illustrator CC (Adobe) software.

### Cyst formation of MDCK cells

MDCK/WT, MDCK/S41A, and MDCK/S41E cells were placed in a 24-well plate (1.0 × 10^5^ cells/well) and cultured for 1 day. These cells were washed twice with PBS (−), detached by trypsinization, and resuspended to single cell suspension in DMEM medium containing 10% fetal bovine serum and 2% Matrigel (356230, Corning) supplemented with or without 3 μg/ml Dox. The cells were placed in a 24-well plate (1.0 × 10^4^ cells/well) precoated with Matrigel and cultured for 5 days with medium change every 2 to 3 days. The resulting cysts were fixed with 4% formaldehyde in PBS (−) for 30 min at room temperature, permeabilized, and blocked at room temperature for 30 min with PBS(−) containing 4% BSA, 0.5% Triton-X100, 0.04% NaN_3_. This solution was also used for dilution of the primary and secondary antibodies. The cysts were incubated with primary antibody for overnight and secondary antibody for 2 h. For DNA and apical membrane staining, cells were incubated with 1.0 μM Hoechst 33342 and 14 nM Actin-Stain 488 Phalloidin (#PHDG1, Cytoskeleton), respectively, together with secondary antibody.

### Western blot analysis

WB was performed as described previously (34, 35). M-phase cells were collected by mitotic shake-off. Whole-cell lysates were prepared using SDS sample buffer containing protease and phosphatase inhibitors [10 μg/ml aprotinin (Fujifilm Wako Pure Chemicals), 4 μg/ml pepstatin A (Peptide Institute, Inc), 10 μg/ml leupeptin (Nacalai Tesque), 2.5 mM EGTA-KOH (Sigma), 1 mM PMSF (Nacalai Tesque), 20 mM β-glycerophosphate (Millipore Sigma), 50 mM NaF (Wako), and 10 mM Na_3_VO_4_ (Wako)]. The proteins were separated by SDS-PAGE and transferred to polyvinylidene difluoride membranes (Pall Corporation). The membranes were incubated with Blocking One (03953-95, Nacalai Tesque) for 1 h at room temperature and incubated with primary and secondary antibodies in 0.1% Tween 20/5% Blocking One/Tris-buffered saline for 1 h at room temperature or overnight at 4 °C. For a peptide-blocking assay, 1.6 μg/ml antibody was incubated with phosphorylated [G(pS)PRVRDDAAFC] or unphosphorylated [GSPRVRDDAAFC] peptides at 250 nM in Tris-buffered saline containing 0.1% Tween 20 on ice at 1 h. Then, these solutions were further diluted ten-fold and used for Western blot analysis. The blots were developed with chemiluminescence reagents (Chemi Lumi-One L, #07880, Chemi-Lumi One Ultra, #11644, Nacalai Tesque; Clarity, 1705061, Bio-Rad), and the signals were detected using a ChemiDoc XRSplus image analyzer (Bio-Rad).

### Cell cycle synchronization

To examine M-phase progression, cells were arrested at the G2/M border by treatment with 5 or 6 μM RO-3306 for 20 h (36, 37, 38). The cells were washed with pre-warmed PBS supplemented with 0.9 mM Ca^2+^ and 0.5 mM Mg^2+^ [PBS (+)] on a water bath at 37 °C. The cells were cultured for another 50 or 60 min without RO-3306, fixed with 2% formaldehyde for 20 min at room temperature, and immunostained for α-tubulin and DNA. Based on the microtubule and chromosome morphologies, M-phase cells were divided into mitotic sub-phases: prophase/prometaphase (P/PM), metaphase (M), anaphase/telophase (A/T), and cytokinesis (Cyt). The mitotic index represents the percentage of the M-phase cells.

To observe the chromosome alignment and localization of RhoG on the plasma membrane, cells were arrested at metaphase as follows. Cells were treated with 5 or 6 μM RO-3306 for 20 h. After a 30-min release from RO-3306, the cells were treated with 40 μM MG132 for 90 min to prevent the onset of anaphase, fixed, and subjected to immunofluorescence staining. For Western blot analysis of metaphase cells, cells were treated with thymidine prior to RO-3306 treatment to increase the efficiency of cell cycle synchronization as follows. Cells were treated with 4 mM thymidine for 24 h and cultured for another 6 h without thymidine after washing the cells four times with pre-warmed PBS (−). The cells were treated with 6 μM RO-3306 for 10.5 h and released from RO-3306 as described above for 30 min. MG132 was added to the cells at 40 μM to prevent the onset of anaphase, and cells were further incubated for 90 min. M-phase cells were collected by mitotic shake-off. For Western blot analysis of cells in M-phase, not just in metaphase, the cells were treated with 5 μM STLC for 16 h, and M-phase cells were collected by mitotic shake-off.

To induce re-expression of WT or mutant Ephexin4, cells capable of Dox-inducible re-expression were treated with 50 ng/ml (HeLa S3/S41E), 75 ng/ml (HeLa S3/S41A) or 90 ng/ml (HeLa S3/WT), 500 ng/ml (HeLa S3/FLAG-RhoG WT), 100 to 700 ng/ml (HeLa S3/FLAG-RhoG G12V), or 25 ng/ml (A549/WT, A549/S41A) Dox together with thymidine until RO3306 treatment.

### Lambda protein phosphatase treatment

STLC-synchronized cells were collected by mitotic shake-off and homogenized with a glass-Teflon homogenizer in ice-cold Hepes-NaOH buffer (pH 7.4, 47 mM) supplemented with 10 μg/ml aprotinin (Fujifilm Wako Pure Chemicals), 4 μg/ml pepstatin A (Peptide Institute, Inc), 10 μg/ml leupeptin (Nacalai Tesque), 1 mM PMSF (Nacalai Tesque), and 9.4% glycerol on ice. After centrifugation at 15,000 rpm for 10 min at 4 °C, the supernatant was incubated with Lambda protein phosphatase (400 units, P0753, New England Biolabs) in 1 × NEBuffer for protein metallophosphatases in the presence of 1 mM MnCl_2_ at 30 °C for 1 h. The reaction mixture was boiled for 5 min with SDS sample buffer supplemented with phosphatase and protease inhibitors [10 μg/ml aprotinin, 4 μg/ml pepstatin A, 10 μg/ml leupeptin, 1 mM PMSF, and 100 mM NaF (Wako)] and analyzed by conventional and Phos-tag SDS-PAGE (20 μM Phos-tag, 40 μM MnCl_2_).

### Measurement of RhoG activity

*E. coli* BL21 cells carrying the GST-ELMO2 plasmid were cultured at 37 °C until the A660 reached 0.8. Protein expression was induced by treatment with 0.3 mM IPTG (099-02534, Wako) at 37 °C for 3 h. Cells were then harvested by centrifugation at 3000 rpm for 10 min. The cells were freeze-thawed and suspended with Hepes-NaOH buffer (pH 7.4, 50 mM) supplemented with 1% Triton X-100, 10% glycerol, 4 mM EDTA-NaOH (pH 7.4), 100 mM NaF, 2 mM PMSF, 2 μg/ml aprotinin, 2 μg/ml leupeptin, 0.8 μg/ml pepstatin A, 0.5 mM EGTA-KOH. Bacterial suspensions were sonicated for 30 s 3 times with 15 s intervals, and the supernatant following centrifugation was stored at −80 °C. The lysate was incubated at 4 °C for 1 h with glutathione sepharose 4B beads (17-0756-01, Amersham Biosciences) pre-equilibrated with Hepes–NaOH buffer (pH 7.4, 50 mM) supplemented with 0.5% Triton X-100, 3.5 mM EGTA–KOH, 10% glycerol, 100 mM NaF, 300 mM NaCl, 10 mM MgCl_2_, 1 mM DTT, 2 mM PMSF, 4 μg/ml aprotinin, 4 μg/ml leupeptin, and 1.6 μg/ml pepstatin A. Then, the beads were washed with Hepes–NaOH buffer used for beads pre-equilibration.

HeLa S3/WT, HeLa S3/S41A, and HeLa S3/S41E cells were treated with Dox at 4 μg/ml, 4 μg/ml, and 3 μg/ml, respectively, for 48 h and then detached from culture plates using PBS supplemented with 0.1% EDTA. After washed with PBS supplemented with 10 mM MgCl_2_, cells were lysed with ice-cold Hepes–NaOH buffer (pH 7.4, 50 mM) supplemented with 1% Triton X-100, 3.5 mM EGTA–KOH, 10% glycerol, 100 mM NaF, 300 mM NaCl, 30 mM MgCl_2_, 1 mM DTT, 2 mM PMSF, 4 μg/ml aprotinin, 4 μg/ml leupeptin, 1.6 μg/ml pepstatin A on ice for 10 min. Cell lysates were centrifuged at 15,000*g* for 15 min, and the supernatants were incubated with GST-ELMO2–bound beads at 4 °C for 1 h. The beads were washed 4 times with Hepes–NaOH buffer used for beads pre-equilibration, and bound proteins were analyzed by Western blot analysis.

### Time-lapse imaging

HeLa S3/WT cells were transfected with siEphexin4 (#3, 250 nM) or siCtrl in a 24-well plate. After 28 h, the cells were treated with 6 μM RO-3306 for 20 h. Immediately after release from RO-3306, time-lapse imaging was performed using a high-content imaging system (Operetta, PerkinElmer Life Sciences) every 5 min for 4 h at 37 °C in 5% CO_2_ as described previously (39). Hoechst 33342 was used to monitor the chromosomes at 0.1 μM.

### Statistics

To analyze significant differences between the two groups, the F test was performed for the determination of variances using Microsoft Excel, followed by unpaired two-tailed Student’s *t* test for two groups with equal variances. For analyses of three or more groups, Bartlett’s test was performed using EZR software (Saitama Medical Center, Jichi Medical University) (40), a graphical user interface for R version 4.3.2 (The R Foundation for Statistical Computing). Tukey’s test and the Games-Howell test were used for groups with equal and unequal variances, respectively.

## Data availability

The data that support the findings of this study are available from the corresponding author upon reasonable request.

## Supporting information

This article contains supporting information.

## Conflict of interest

The authors declare that they have no conflicts of interest with the contents of this article.
