# Supplementary material for: Phosphorylation of Ephexin4 at Ser-41 contributes to chromosome alignment via RhoG activation in cell division
Source: J Biol Chem. 2024 Dec 13;301(1):108084. doi: 10.1016/j.jbc.2024.108084 (PMC11758948; doi:10.1016/j.jbc.2024.108084)
Supplement: Supporting information [file mmc1.pdf]

Supporting Information

Phosphorylation of Ephexin4 at Ser-41 contributes to chromosome alignment via RhoG activation in cell division

Ryuji Yasutake[1], Hiroki Kuwajima[1], Ryuzaburo Yuki[1], Junna Tanaka[1], Youhei Saito[1], Yuji Nakayama[1, *]

[1]Laboratory of Biochemistry and Molecular Biology, Kyoto Pharmaceutical University, Kyoto 607-8414, Japan

Running title: Ephexin4 phosphorylation regulates cell division

*Corresponding author: Yuji Nakayama, Ph.D.
Laboratory of Biochemistry & Molecular Biology
Kyoto Pharmaceutical University
5 Misasagi-Nakauchi-cho, Yamashina-ku, Kyoto 607-8414, Japan
Phone: +81-75-595-4653; Fax: +81-75-595-4758
Email: nakayama@mb.kyoto-phu.ac.jp

Supplementary Figure 1

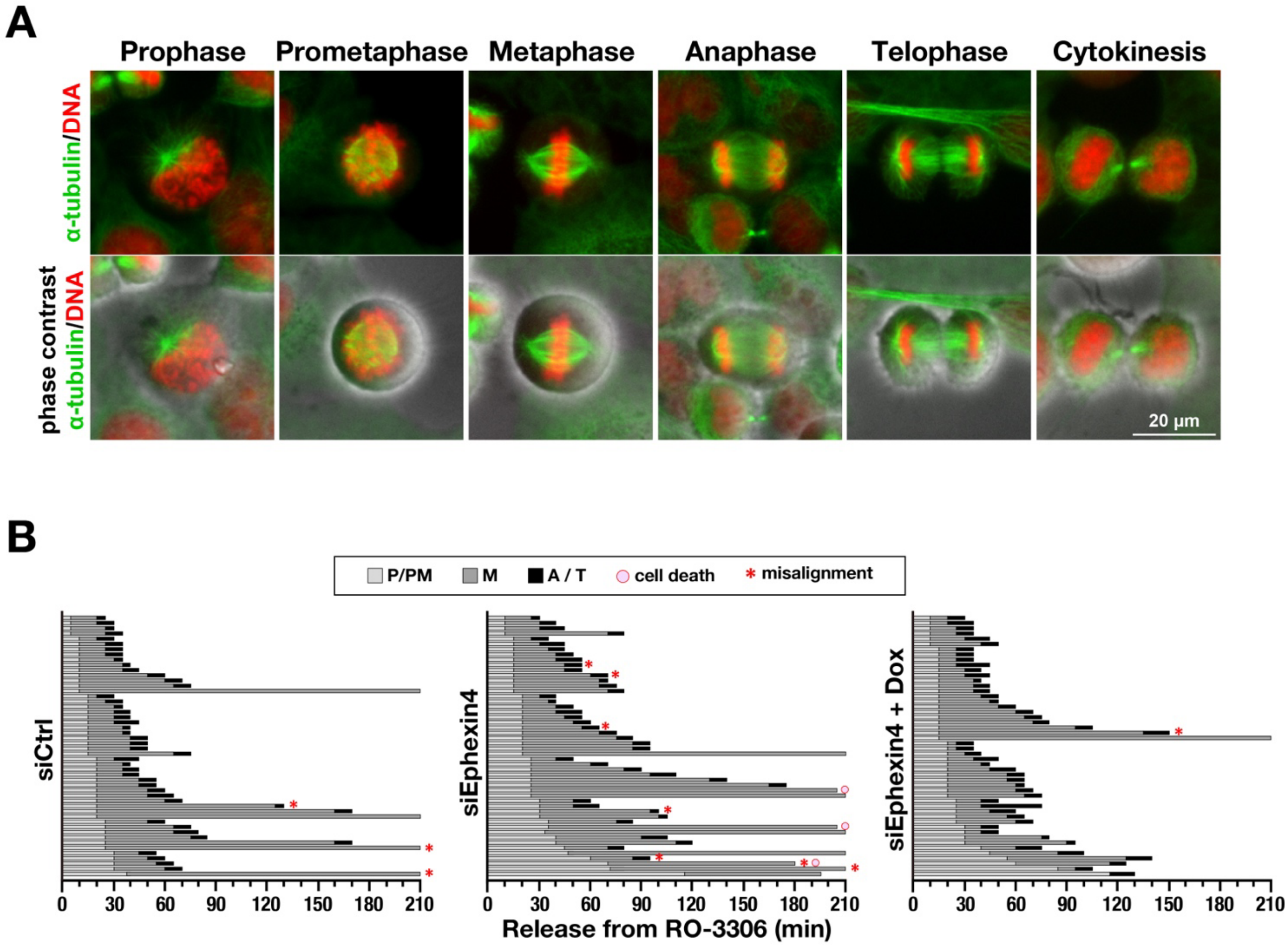

Figure S1. Ephexin4 is necessary for the plasma membrane localization of RhoG and chromosome alignment.
A. Typical images of mitotic sub-phase are shown. *B*. The duration of each mitotic phase of each cell in Figure 1*E* is shown: prophase/prometaphase (P/PM, pale gray), metaphase (M, gray), and anaphase/telophase (A/T, black). Cell death (circle) and chromosome misalignment (asterisk) are shown in the graph. The experiments were independently performed three times and a representative result is shown.

Supplementary Figure 2

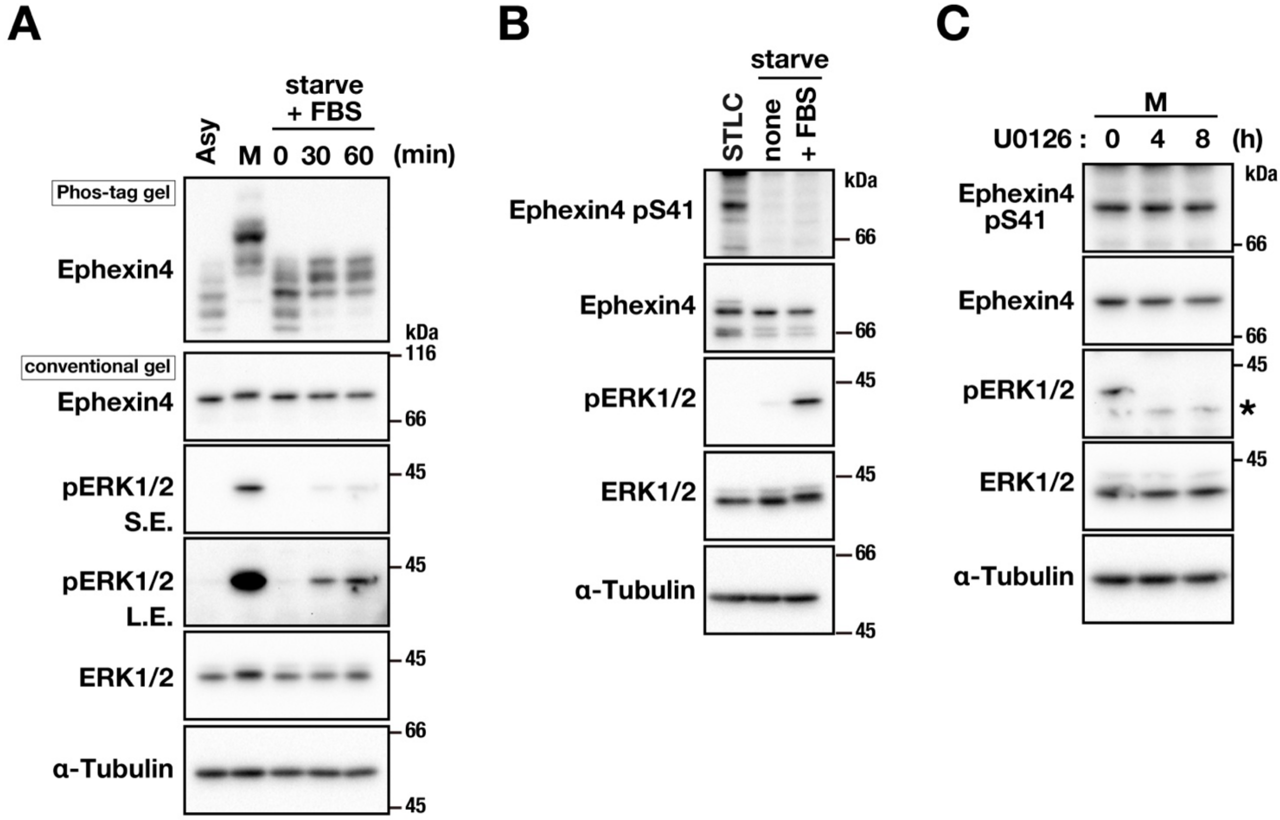

Figure S2. Ephexin4 phosphorylation patterns in M phase differ from those in interphase, even upon serum stimulation.

Interphase cells with or without serum stimulation after starvation and M-phase cells were collected, and whole cell lysates were subjected to western blot analysis using Phos-tag gel (*A*) or conventional gel (*A*, *B*) for SDS-PAGE. *A*. HeLa S3 cells were treated with 5 μM STLC for 16 h, and M-phase cells were collected by mitotic shake-off. For serum stimulation, HeLa S3 cells were cultured without fetal bovine serum (FBS) for 24 h and then cultured in fresh medium supplemented with 10% FBS for 30 or 60 min. *B*. hTERT RPE-1 cells were cultured without FBS for 24 h and then cultured with or without FBS for 1 h. M-phase cells were collected as described in *A*. *C*. HeLa S3 cells were treated with 5 μM STLC for 8 h with or without 10 μM U0126 (0, 4 h for the second half, 8 h). M-phase cells were collected by mitotic shake-off.

Supplementary Figure 3

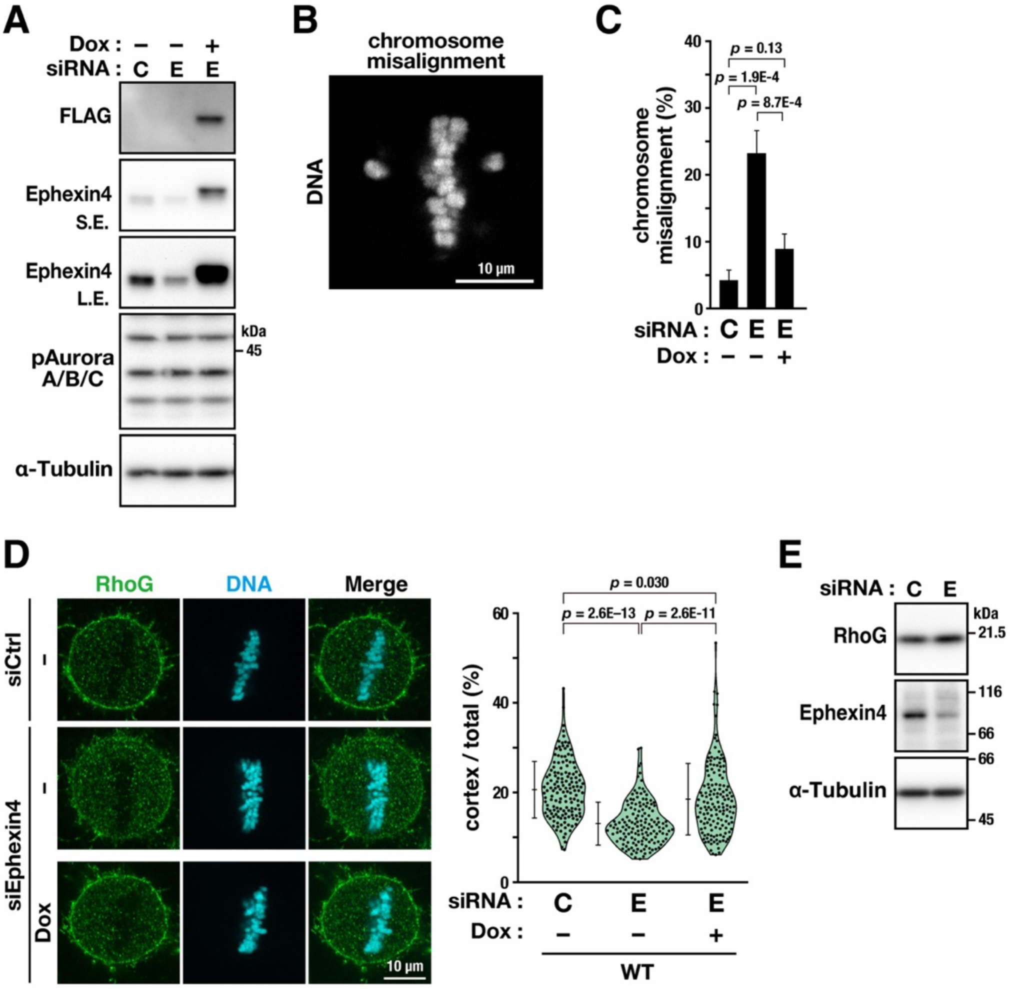

Figure S3. Ephexin4 is necessary for the plasma membrane localization of RhoG and chromosome alignment. *A*, HeLa S3/WT cells were transfected with Ephexin4-targeting (E, #1, 1.25 nM) or non-targeting (C, 1.25 nM) siRNA. After 7.5 h of transfection, the cells were treated with 4 mM thymidine for 24 h and cultured without thymidine for 6 h. Then, the cells were treated with 6 μM RO-3306 for 10.5 h. After a 30-min release from RO-3306, the cells were treated with 40 μM MG132 for 90 min. For Dox-inducible expression, cells were continuously treated with 90 mg/ml Dox from the siRNA transfection until end of RO-3306 treatment. M-phase cells were collected by mitotic shake-off, and whole-cell lysates were subjected to western blot analysis. *B–D*, HeLa S3/WT cells were transfected with Ephexin4-targeting (E, #1, 1.25 nM) or non-targeting (C, 1.25 nM) siRNA and cultured for 48 h with or without 90 ng/ml Dox. During the last 20 h, the cells were treated with 6 μM RO-3306. After a 30-min release from RO-3306, the cells were treated with 40 μM MG132 for 90 min and fixed for DNA and RhoG staining. Representative image of chromosome misalignment is shown in *B*. In *C*, the percentage of cells exhibiting chromosome misalignment is shown as the mean ± SD of three independent experiments (WT, n ≥ 34 cells per condition). Tukey's test was used to determine *p*-values. In *D*, fluorescence intensity of RhoG at the plasma membrane and that in the whole-cell area were quantified in metaphase cells. The ratio of RhoG at the plasma membrane versus the whole-cell area is plotted as the mean ± SD of three independent experiments (WT, n ≥ 34 cells per condition). The Games–Howell test was used to determine *p*-values. Scale bar, 10 μm. *E*, HeLa S3 cells were transfected with Ephexin4-targeting (E, #1, 1.25 nM) or non-targeting (C, 1.25 nM) siRNA and cultured for 48 h. During the last 20 h, the cells were treated with 6 μM RO-3306. After a 30-min release from RO-3306, the cells were treated with 40 μM MG132 for 90 min, and whole-cell lysates were subjected to WB analysis.

Supplementary Figure 4

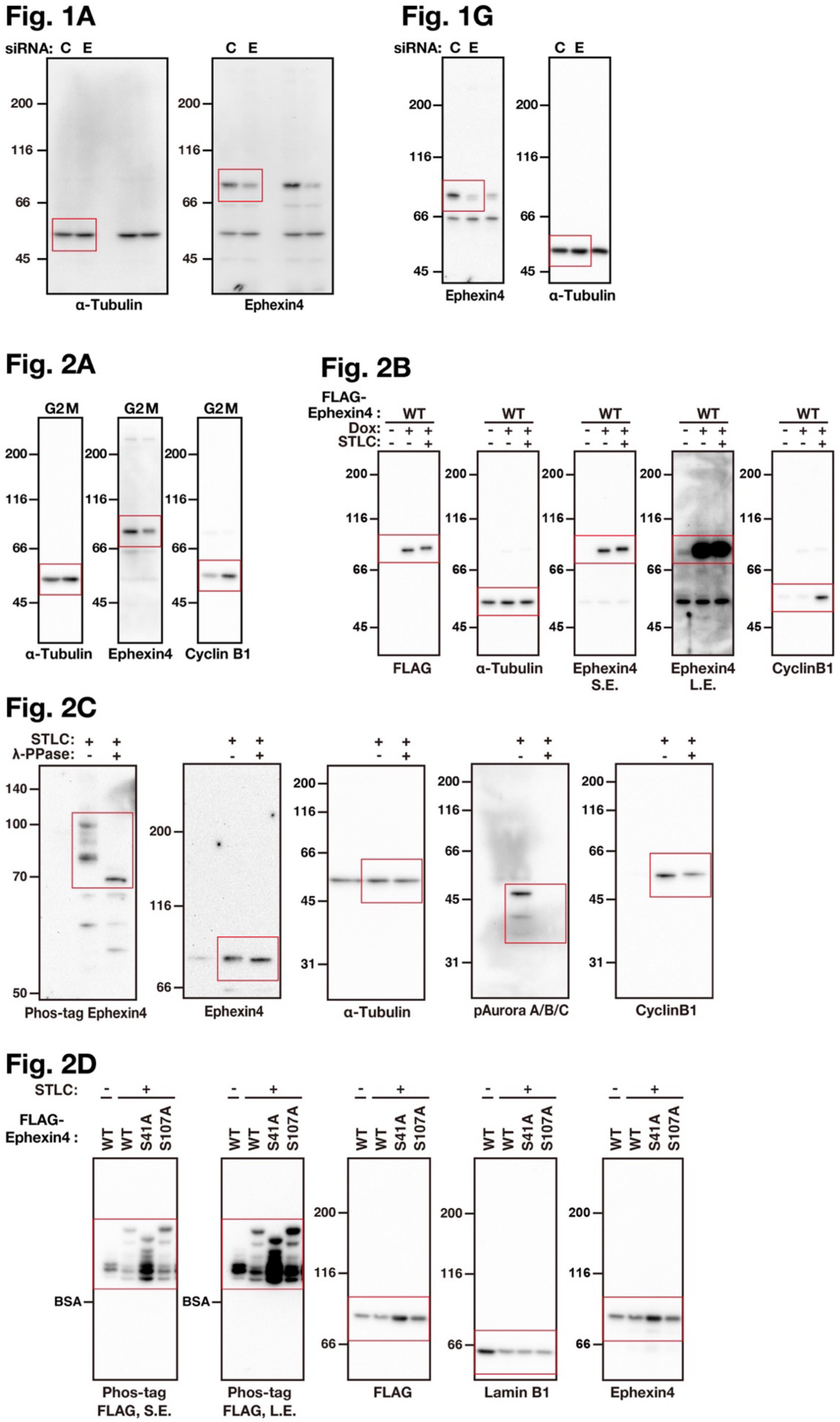

Figure S4. Full-length blots for Figures 1 and 2.

Supplementary Figure 5

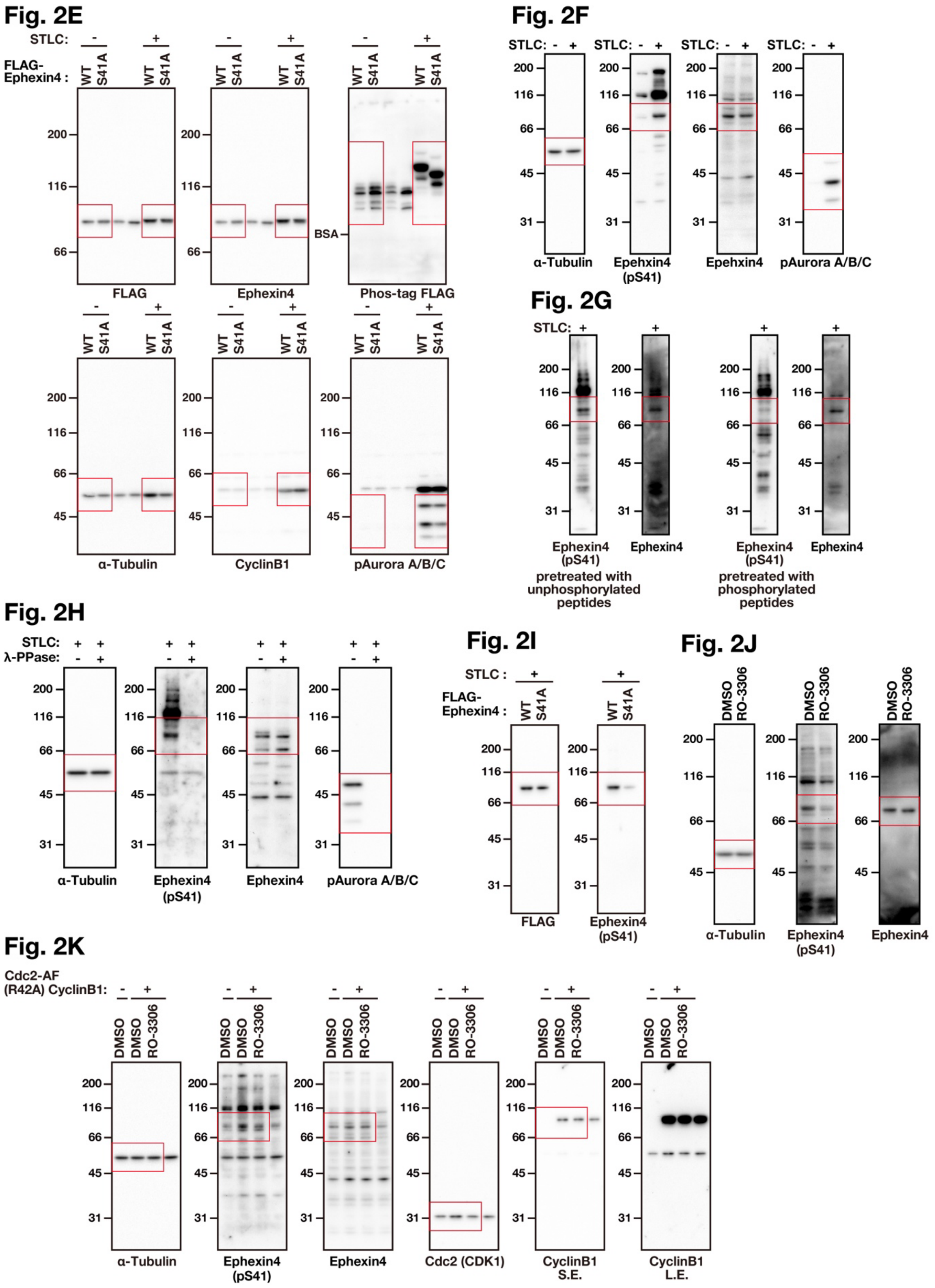

Figure S5. Full-length blots for Figure 2.

Supplementary Figure 6

Fig. 3A

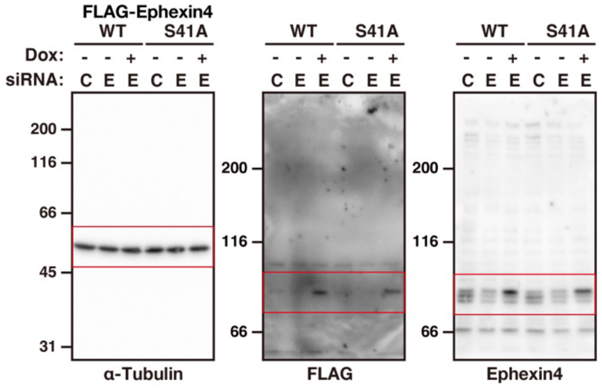

Fig. 4A

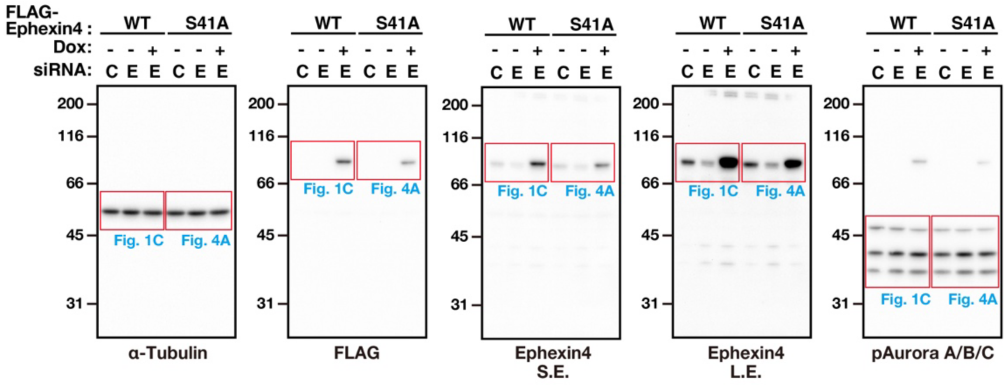

Fig. 4A

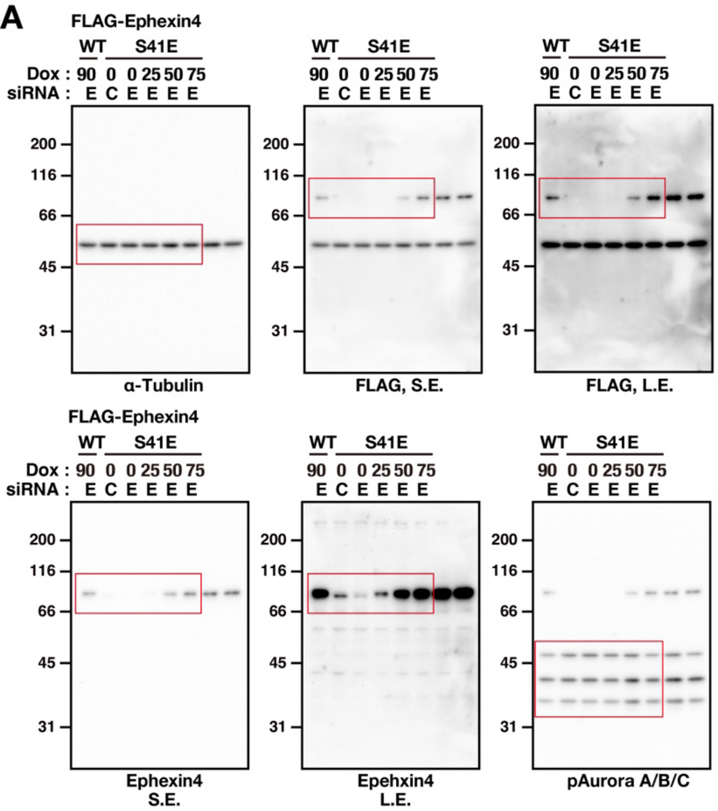

Figure S6. Full-length blots for Figures 3 and 4.

Supplementary Figure 7

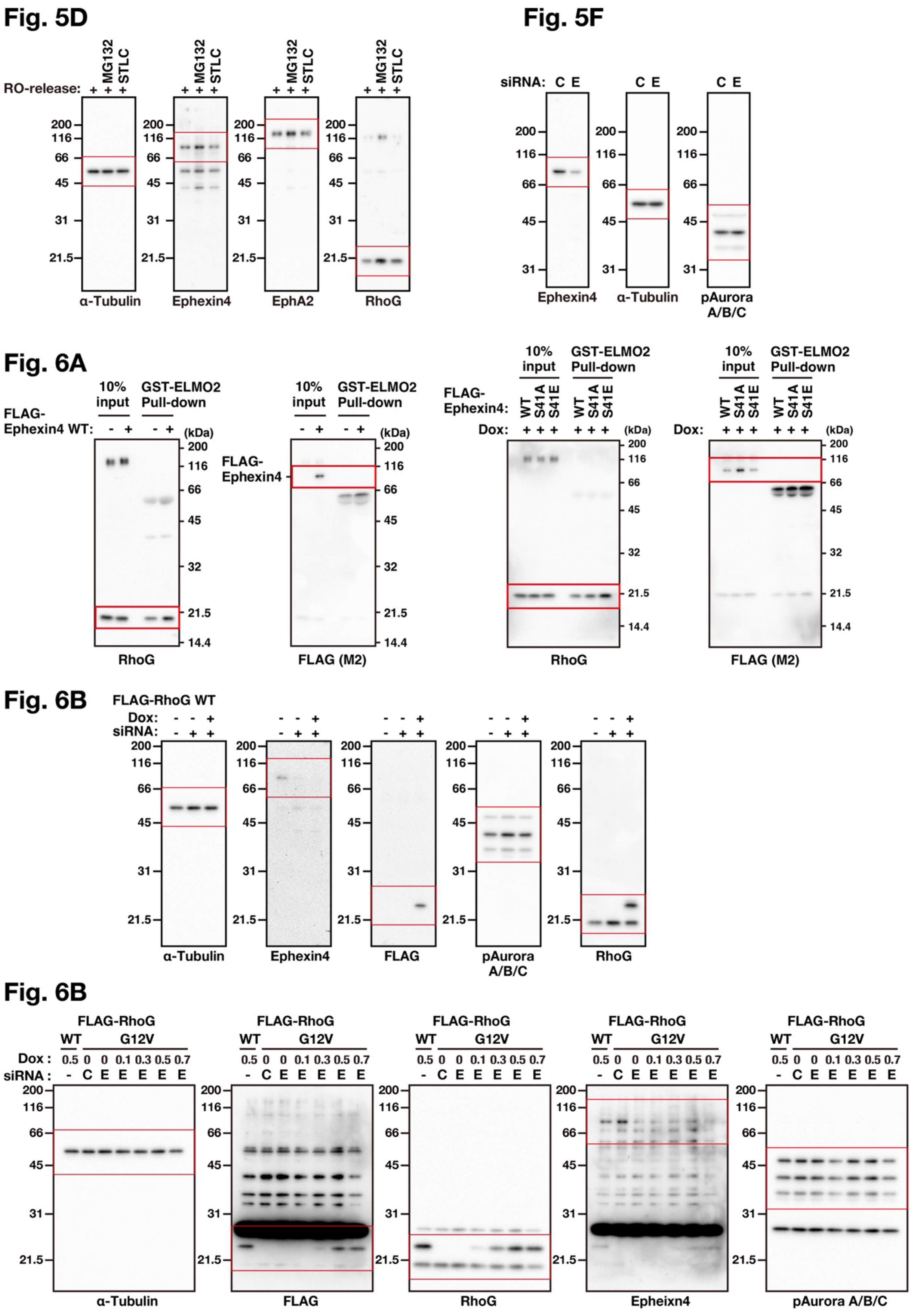

Figure S7. Full-length blots for Figures 5 and 6.

Supplementary Figure 8

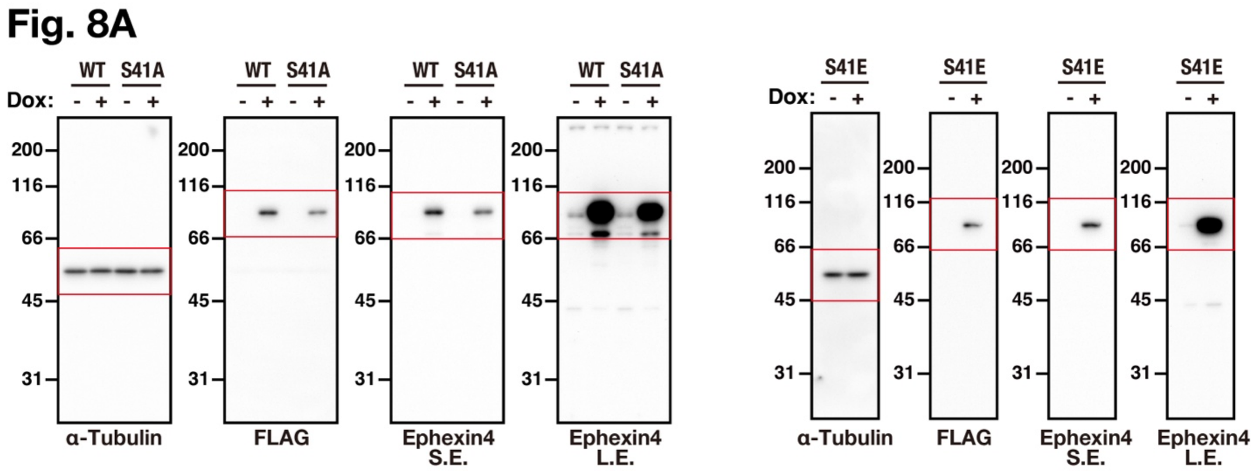

Figure S8. Full-length blots for Figure 8.

Supplementary Figure 9

Fig. S2A

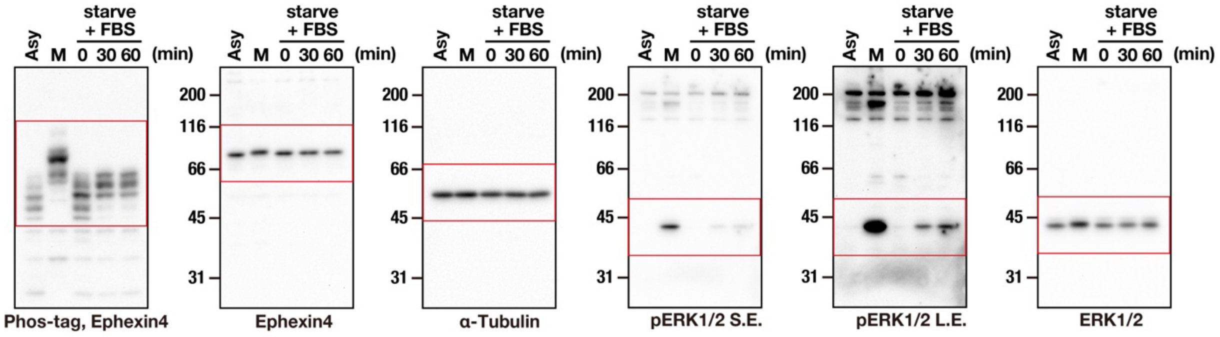

Fig. S2B

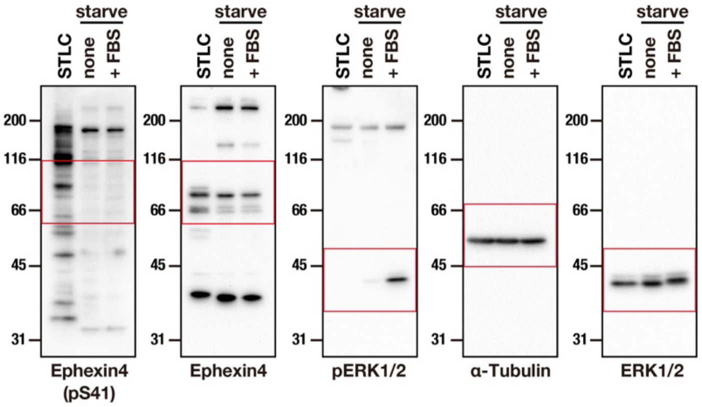

Fig. S2C

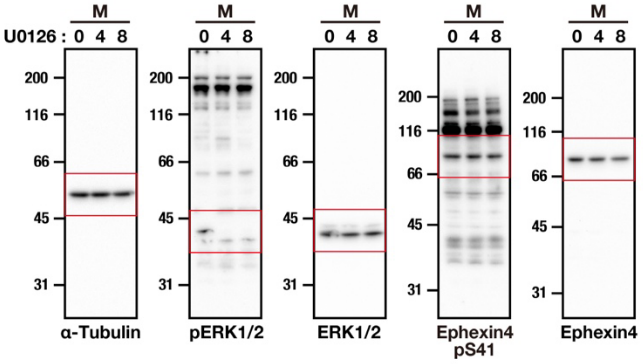

Fig. S3E

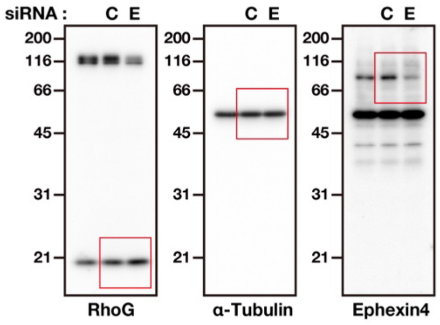

Fig. S3A

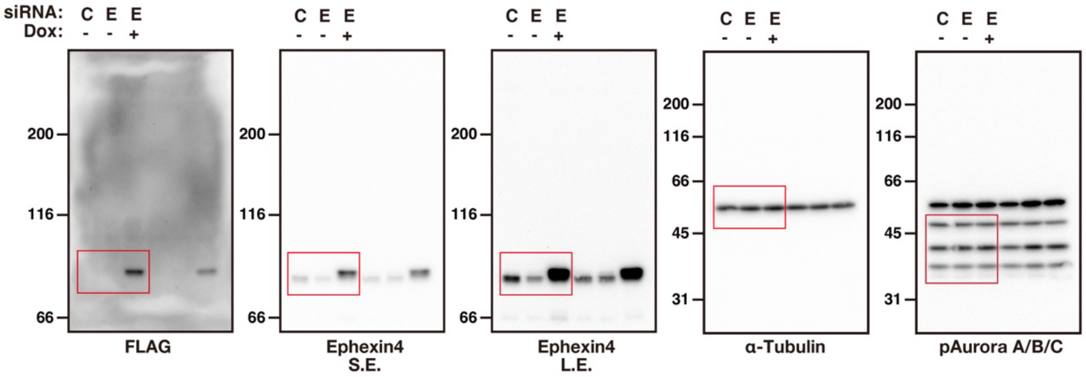

Figure S9. Full-length blots for Figures S2 and S3.
